# Alkaline pH Promotes NADPH Oxidase-Independent Neutrophil Extracellular Trap Formation: A Matter of Mitochondrial Reactive Oxygen Species Generation and Citrullination and Cleavage of Histone

**DOI:** 10.3389/fimmu.2017.01849

**Published:** 2018-01-09

**Authors:** Cristiane Naffah de Souza, Leandro C. D. Breda, Meraj A. Khan, Sandro Rogério de Almeida, Niels Olsen Saraiva Câmara, Neil Sweezey, Nades Palaniyar

**Affiliations:** ^1^Program in Translational Medicine, Peter Gilgan Centre for Research and Learning, The Hospital for Sick Children, Toronto, ON, Canada; ^2^Department of Laboratory Medicine and Pathobiology, Faculty of Medicine, The University of Toronto, Toronto, ON, Canada; ^3^Department of Immunology, Institute of Biomedical Sciences, University of São Paulo, Butantã, Brazil; ^4^Department of Clinical and Toxicological Analyses, School of Pharmaceutical Sciences, University of São Paulo, Ribeirão Preto, Brazil; ^5^Institute of Medical Sciences, University of Toronto, Toronto, ON, Canada

**Keywords:** NET, NOX-independent, pH, calcium, mitochondrial ROS, PAD4, histone 3 citrullination, histone cleavage

## Abstract

pH is highly variable in different tissues and affects many enzymatic reactions in neutrophils. In response to calcium ionophores such as A23187 and ionomycin, neutrophils undergo nicotinamide adenine dinucleotide phosphate oxidase (NOX)-independent neutrophil extracellular trap (NET) formation (NETosis). However, how pH influences calcium-dependent Nox-independent NET formation is not well understood. We hypothesized that increasing pH promotes Nox-independent NET formation by promoting calcium influx, mitochondrial reactive oxygen species (mROS) generation, histone citrullination, and histone cleavage. Here, we show that stimulating human neutrophils isolated from peripheral blood with calcium ionophore A23187 or ionomycin in the media with increasing extracellular pH (6.6, 6.8, 7.0, 7.2, 7.4, 7.8) drastically increases intracellular pH within in 10–20 min. These intracellular pH values are much higher compared to unstimulated cells placed in the media with corresponding pH values. Raising pH slightly drastically increases intracellular calcium concentration in resting and stimulated neutrophils, respectively. Like calcium, mROS generation also increases with increasing pH. An mROS scavenger, MitoTempo, significantly suppresses calcium ionophore-mediated NET formation with a greater effect at higher pH, indicating that mROS production is at least partly responsible for pH-dependent suppression of Nox-independent NETosis. In addition, raising pH increases PAD4 activity as determined by the citrullination of histone (CitH3) and histone cleavage determined by Western blots. The pH-dependent histone cleavage is reproducibly very high during ionomycin-induced NETosis compared to A23187-induced NETosis. Little or no histone cleavage was noted in unstimulated cells, at any pH. Both CitH3 and cleavage of histones facilitate DNA decondensation. Therefore, alkaline pH promotes intracellular calcium influx, mROS generation, PAD4-mediated CitH3 formation, histone 4 cleavage and eventually NET formation. Calcium-mediated NET formation and CitH3 formation are often related to sterile inflammation. Hence, understanding these important mechanistic steps helps to explain how pH regulates NOX-independent NET formation, and modifying pH may help to regulate NET formation during sterile inflammation or potential damage caused by compounds such as ionomycin, secreted by *Streptomyces*, a group of Gram-positive bacteria well known for producing antibiotics.

## Introduction

Neutrophils are the most abundant leukocytes in human peripheral blood, consisting approximately 50–70% of white blood cells. Their functions include phagocytosis, granule release, and neutrophil extracellular trap (NET) formation or NETosis. NET formation was discovered in 1996, and the study of NETosis has been very active after showing that NETs could kill bacteria, in 2004 (1, 2). To date, two distinct types of NETosis have been established: NOX-dependent and NOX-independent pathways (2–4). NOX-independent pathway does not require NOX-mediated reactive oxygen species (ROS) production; instead, mitochondrial reactive oxygen species (mROS) generation occurs in this pathway (5). Compared to NOX-dependent NETosis, NOX-independent NETosis induced by calcium ionophores A23187 and ionomycin is rapid (4, 5). Peptidylargininedeiminase 4 (PAD4), an enzyme that catalyzes protein citrullination, plays a key role in NOX-independent NETosis (6, 7). Once bound to calcium, PAD4 present in the cytosol translocates into the nucleus, where it deiminates positively charged arginine present on histones to non-charged citrulline, facilitating chromatin decondensation, particularly at promoter regions (6).

pH regulates the activities of several enzymes in cells, including neutrophils. PAD4 has an alkaline pH optimum (~7.6–8.0) (8); therefore, this enzyme is expected to be more active at alkaline pH. Neutrophil elastase (NE) cleaves histone H4 (9) that is also considered to help chromatin decondensation during NET formation. The pH optimum for NE is also alkaline (~8.0–8.5) (10). Hence, these are good candidate enzymes that could help promote NET formation at higher pHs.

Several studies have demonstrated that lowering intracellular pH leads to an impaired neutrophil function (11). The effect of pH on NET formation is beginning to be examined (12, 13). Nevertheless, how pH affects various steps of NET formation is still not completely understood. Particularly, it is unclear whether the pH interferes with calcium influx, mROS production and histone cleavage in neutrophils. Therefore, we aimed to understand the regulatory mechanism of alkalinization (increased pH) on NOX-independent NETosis. Our studies show that extracellular pH rapidly affects the intracellular pH, and raising pH increases calcium influx, mROS generation, PAD4 activity and histone 4 cleavage, and consequently promotes NET formation. Taken together, our findings help to better understand the molecular mechanism of NOX-independent NETosis and suggest the potential of modifying pH to regulate NET formation at the sites of inflammation.

## Materials and Methods

### Research Ethics Board Approval

The study protocol for using human blood samples was approved by the ethics committee of The Hospital for Sick Children, Toronto. All the procedures including healthy human volunteer recruitment for blood donation were performed in accordance with the ethics committee guidelines. All the volunteers participating in this study gave their signed consent prior to the blood donation.

### Primary Human Neutrophils

Peripheral blood from healthy male donors were drawn in K2 EDTA blood collection tubes (Becton, Dickinson and Co.) in the hospital at nursing station. The neutrophils isolation was performed using PolymorphPrep (Axis-Shield), according to the company protocol (as previously reported). Briefly, equal volume of blood was laid over PolymorphPrep solution and centrifuged for 35 min at room temperature without applying breaks. After centrifugation, the polymorphonuclear neutrophil layer was collected and washed with washing solution [0.425% (w/v) NaCl with 10 mM HEPES] to eliminate all the residues of PolymorphPrep. Then, red blood cells were lysed twice with 0.2% (w/v) NaCl hypotonic solution for 30 s followed by adding an equal volume of 1.6% (w/v) NaCl solution with 20 mM HEPES buffer to obtain the isotonic condition. After lysis, two more washes were done to eliminate red blood cells debris and soluble components. Neutrophils were resuspended in RPMI medium (Invitrogen) containing 10 mM HEPES (pH 7.2) and counted using hemocytometer. Viability of the purified neutrophils were checked by Trypan blue exclusion assay. Neutrophils purity was determined by imaging Cytospin preparations. Only neutrophil preparations having more than 95–98% viability and purity were used in the experiments. Cells were kept at 37°C and 5% (v/v) CO_2_ incubator for the entire experimental period.

### Media Preparation to Change pH

The volume of HCl (5 M) or NaOH (5 M) necessary to modify the initial media pH (7.2) to 6.6, 6.8, 7.0, 7.2, 7.4, 7.6, and 7.8 was predetermined using a pH meter. The isotonic RPMI media with different pHs were added to the neutrophil suspension for further experiments. Each experiment performed in this study had a technical duplicate, and the assay was repeated with different donors to obtain biological replicates (*n* = 3–5; specific details are given in the figure legends).

### Sytox Green NET Formation Assay

The NETotic index was measured by Sytox Green, a cell-impermeable DNA dye (Life Technologies), fluorescence. Briefly, neutrophils were resuspended at 1 × 10^6^ cells per mL in RPMI media (pH 7.2), containing 5 µM of Sytox Green dye. Later, 50,000 neutrophils were seeded into 96-well plates containing 50 µL of media with predetermined pH to adjust the pH of corresponding wells. The cells were stimulated with NOX-independent agonists calcium ionophores A23187 (4 µM) or ionomycin (5 µM) and kept at 37°C and 5% (v/v) CO_2_ incubator. Sytox Green fluorescence intensities were detected by a POLARstar OMEGA fluorescence microplate reader (BMG Labtech) every 30-min intervals for 240 min. The plates were briefly (~2–5 min) taken out of the incubator for the readings. The NETotic index was calculated based on the value of 100% NET formation obtained by lyzing the cells with 0.5% (v/v) Triton X-100 (representing total DNA presenting in the sample). The baseline green fluorescence at time 0-min was subtracted from the fluorescence at each time point and was then divided by the fluorescence values of cell lysed with Triton X-100. To determine whether pH affects SYTOX Green fluorescence, the same number of cells were lyzed with Triton in various pH buffers, and the green fluorescence of 5 µM SYTOX Green was recorded every 30 min for 240 min.

### Changes in Intracellular pH

We used the SNARF^®^-4F 5-(and-6)-carboxylic acid (Thermo Fisher Scientific) dye to determine the changes in intracellular pH after adjusting extracellular pH. First, cells were preloaded with 10 µM dye for 30 min at 37°C and 5% (v/v) CO_2_, washed and resuspended in fresh RPMI (pH 7.2) at a concentration of 1 × 10^6^ cells/mL. Neutrophils (0.5 × 10^5^ cells in 50 µL) were seeded into a 96-well plate containing the same volume of media to adjust the pH to different values (6.6, 6.8, 7.0, 7.2, 7.4, 7.6, and 7.8). These neutrophils were stimulated either with only media (negative control), A23187 (4 µM; Sigma-Aldrich) or ionomycin (4 µM; Sigma-Aldrich) and dual emission spectra of SNARF were measured. pH changes were recorded every 10 min up to 60 min. The emission spectrum of SNARF undergoes a pH-dependent wave length shift; therefore, the ratio (580/640 nm) of the fluorescence intensities from the dye at two emission wavelengths were used for intracellular pH determinations. Carboxy SNARF-4F is typically used by exciting the dye at one wave length (between 488 and 530 nm), while monitoring the fluorescence emission at two wave lengths (580 and 640 nm). The ratio of the fluorescence intensities of two emission wavelengths 640/580 nm were used as a proxy for intracellular pH. Under this format, increasing SNARF ratio reflects higher pH.

### Intracellular Calcium Levels

Intracellular calcium concentrations were assessed using Fluo-4-AM calcium indicator dye by a plate reader assay. Briefly, 1 × 10^6^ cells per mL were incubated in HBSS-Mg^2+^ (calcium free) media with 4 µM Fluo-4-AM for 15 min at 37°C and 5% (v/v) CO_2_. After washing, the cells were resuspended in RPMI (pH 7.2) and 50,000 neutrophils (50 µL) were seeded in a well of 96-well plates containing 50 µL of media to adjust the pH of corresponding wells. Directly after seeding the cells, Fluo-4-AM fluorescence was measured using the SpectraMax Gemini EM fluorescence microplate reader (0-min; Molecular Devices). Cells were incubated for 10 min at 37°C to allow intracellular pH changes. After incubation, Fluo-4-AM fluoresce was measured. Cells were then stimulated with either calcium ionophore A23187 or ionomycin. After adding calcium ionophores or media controls, the Fluo-4-AM fluorescence intensities were recorded every 30 s up to 1,200 s (at 37°C). The plates were not taken out from the microplate reader until the end of experiment. The ratio comparing each time point with the 0-min reading was calculated and used for determining the calcium influx. To determine whether pH affects Fluo-4 AM fluorescence, the experiment was repeated with media containing Fluo-4 with no cells.

### mROS Detection

To detect the mROS production, a plate reader assay was performed using the MitoSOX probe. Briefly, 1 × 10^6^ cells per mL were incubated with 4 µM of MitoSOX Red for 15 min at 37°C and 5% CO_2_ and seeded in a black clear bottom 96-well plate containing 50 µL of specific media to adjust the pH of corresponding wells. The cells were stimulated with 4 µM of calcium ionophore A23187 or ionomycin, and the fluorescence was measured every 4 min up to 120 min using the SpectraMax Gemini EM fluorescence microplate reader (Molecular Devices). To determine whether pH affects 4 µM MitoSox fluorescence, the experiment was repeated with media containing MitoSox with no cells.

### Immunofluorescence Confocal Imaging

The same method described above was used for obtaining the pH 6.6, 7.2, or 7.8 used in the immunofluorescence. A total of 100,000 neutrophils were seeded into 12-well chamber slides at 37°C (Ibidi, cat #81201) and the cells were activated with either media only (negative control), 4 µM of calcium ionophore A23187 or ionomycin for 30 min (PAD4 and Citrullinated Histone 3 staining) or 120 min (MPO and Citrullinated Histone 3 staining). The cells and NETs were fixed with paraformaldehyde [4% (w/v) for 15 min], permeabilized for 15 min with 0.1% Triton-X 100, and blocked with 5% (w/v) BSA for 1 h. The following antibodies were used for immunostaining: mouse anti-PAD4 antibody (ab128086, Abcam; 1:100 dilution) or mouse anti-myeloperoxidase antibody (ab25989, Abcam; 1:400 dilution) was used for staining PAD4 and MPO, respectively (with secondary antibody conjugated with a green fluorescence Alexa fluor 488 dye; 1:5,000 dilution; Thermo Fisher Scientific), while rabbit anti-citrullinated histone 3 antibody (ab5103, Abcam; 1:400 dilution) was used for detecting the presence of CitH3 (with secondary antibody conjugated with a far red fluorescence dye Alexa fluor 647; 1:5,000 dilution; Thermo Fisher Scientific). DAPI was used to stain the DNA (1:100 dilution). As isotype controls, we used Mouse IgG (eBioscience 14-4714-81; 1:100 dilution) and Rabbit IgG (Invitrogen; 31235; 1:100 dilution). After treating with the secondary antibody, slides were washed and mounted by glass cover slips (Fisher Scientific) with anti-fade fluorescent mounting medium (Dako). The images were then taken using an Olympus IX81 inverted fluorescence microscope with a Hamamatsu C9100-13 back-thinned EM-CCD camera and Yokogawa CSU × 1 spinning disk confocal scan head with Spectral Aurora Borealis upgrade, four separate diode-pumped solid-state laser lines (Spectral Applied Research, 405, 491, 561, and 642 nm). The images were taken at 40×/0.95 magnification and processed by Velocity software (version 6.3, Cell Imaging Perkin-Elmer).

### Immunoblotting

Tubes containing 0.5 × 10^6^ neutrophils in pH adjusted (6.6, 7.2, or 7.8) media were activated either by negative control (only media) or by calcium ionophore A23187 or ionomycin for 60 min (at 37°C and 5% CO_2_ incubator). After incubation, cells were lysed using the lysis buffer containing 1% (w/v) Triton X-100, 25 mM NaF, 50 mM Tris, 10 mM KCl, 10 µg/mL Aprotinin, 2 mM PMSF, 1 mM Levanisole, 1 mM NaVO_3_, 0.5 µM EDTA, 25 µM Leupeptin, 25 µM Pepstatin, 1 protease inhibitor cocktail tablets per 5 mL (Roche), and 1 phosphatase inhibitor cocktail tablet per 10 mL (Roche). A quarter volume of 5 × loading dye [125 mM Tris.HCl at pH 6.8, 6% (w/v) SDS, 8% (v/v) β-mercaptoethanol, 18% (v/v) glycerol, and 5 mM EDTA, 5 mM EGTA] was added followed by 10 min of heating at 95°C with 350 rcm shaking. The samples were separated in a 5% (w/v) stacking and 10% (w/v) resolving gel at 100 V and transferred on a nitrocellulose membrane for 70 min at 350 mA. After transfer, the membranes were blocked with 5% (w/v) BSA in 0.05% PBST for 2 h at room temperature. The membranes were incubated in the primary antibody at 4°C overnight followed by three washes with PBS with 0.1% Tween (PBST) for 30 min. The antibodies used were: anti-Histone H4 (ab16483, Abcam; rabbit pAb; 1:1,000) and anti-GAPDH (FL-335, Santa Cruz; rabbit pAb; 1:5,000). The membranes were then incubated with the secondary antibody [donkey anti-rabbit IgG-HRP (31458, Thermo Fisher; 1:10,000)] for 1 h and then washed three times with 0.1% PBST for 30 min. The densitometry analysis of the blots was done using the Image Studio software (LI-COR Biotechnology) and normalized to GAPDH.

### Statistical Analysis

All statistical analyses were performed on GraphPad Prism 7. One-way ANOVA with Dunnett and Tukey’s post-tests, two-way ANOVA with Bonferroni posttest or *t*-test was done as appropriate. A *p*-value of less than 0.05 was considered to represent significant differences between conditions. All the data are presented as mean ± SEM.

## Results

### Alkaline pH Promotes Calcium Ionophore-Induced NET Formation

**Figure 1:**
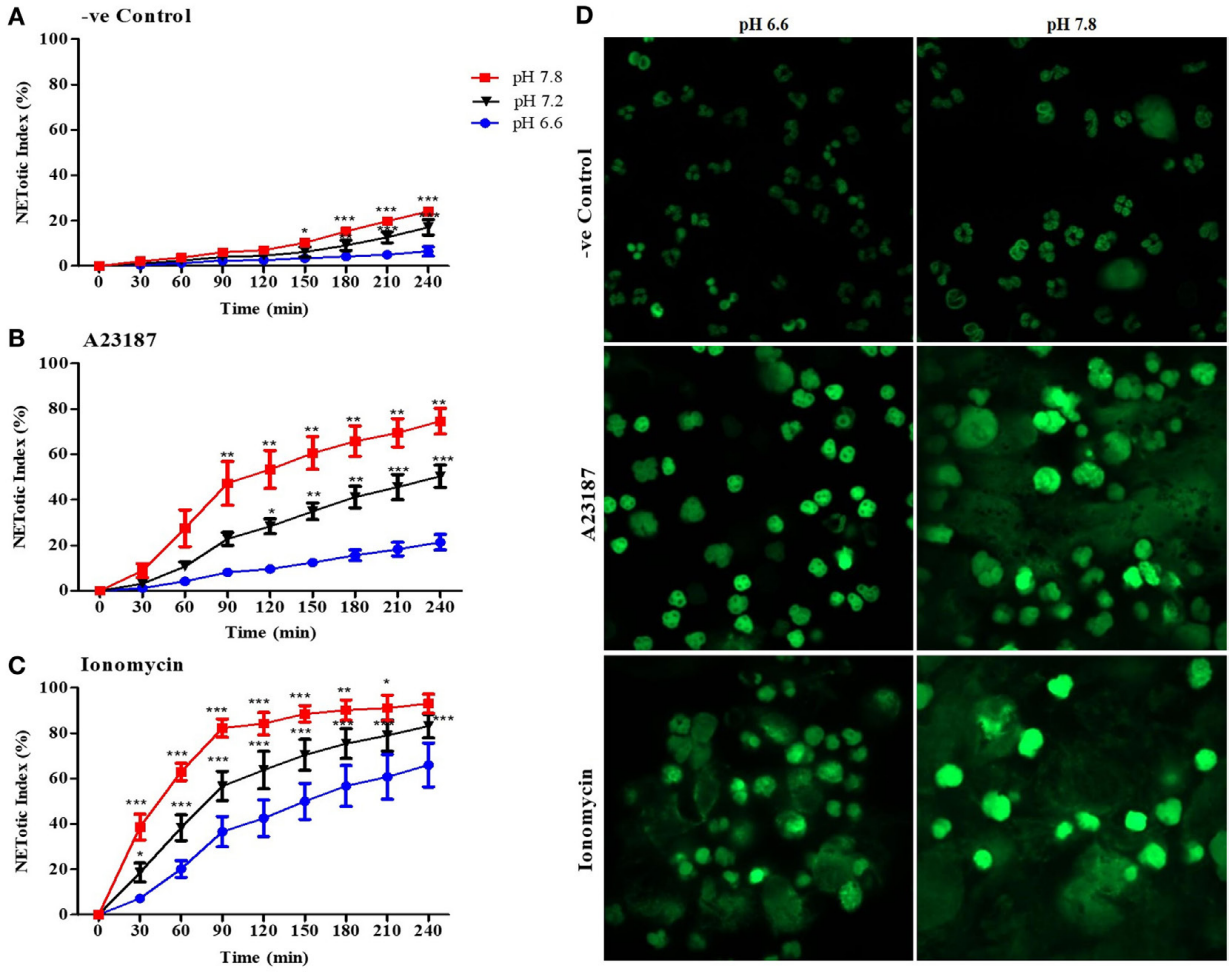
Higher pHs increase spontaneous and A23187 or ionomycin-mediated neutrophil extracellular trap (NET) formation. Neutrophils resuspended in media of predetermined extracellular pH (6.6, 7.2, and 7.8) containing 5 µM Styox Green dye in resting condition (negative control). Florescence was recorded by a plate reader for every 30 min up to 4 h. % DNA release (NETotic index) shows more NETosis in higher extracellular pH conditions, in resting neutrophils (negative control) **(A)**, and after stimulation with A23187 **(B)** or ionomycin **(C)**. **(D)** Neutrophils were resuspended in RPMI media, containing 5 µM of SYTOX Green dye, at two different pHs (6.6 and 7.8) and seeded in a chamber slide for 4 h with or without A23187 or ionomycin. After incubation, cells were fixed with 4% (v/v) PFA for 15 min and analyzed by confocal microscopy. The images clearly show a greater fluorescence at pH 7.8 **(D)**. *n* = 3–5. SYTOX Green DNA dye-green (scale bar 22 µm). *n* = 5, two-way ANOVA with Bonferroni’s post-test. *p < 0.05, **p < 0.01, ***p < 0.001. See Figure S1 in Supplementary Material for the NET formation assay in pHs 6.6, 6.8, 7.0, 7.2, 7.4, 7.6, and 7.8 and the regression slope of the NET formation at 120- and 240-min time points at different pH. See Figure S2 in Supplementary Material, which indicates that pH does not directly affect SYTOX Green florescence intensity. See Table S1 in Supplementary Material for the SYTOX assay in earlier time points.

To determine the effect of pH on NOX-independent NETosis, we incubated purified peripheral blood neutrophils in media with seven different pHs (6.6–7.8) in the absence (negative control) or presence of calcium ionophores A23187 or ionomycin. The percentage DNA release in each condition was calculated by comparing the green fluorescent intensity of the cells at specific conditions with the cells treated with Triton (100% DNA release). The percentage DNA release (or NETotic index) calculated by SYTOX Green fluorescence showed that alkaline and acidic pHs increased and decreased NET formation, respectively (Figures 1A–C; Figures S1A–C in Supplementary Material; pH itself does not affect SYTOX Green fluorescence signal, Figure S2 in Supplementary Material). A regression line at 120 and 240-min time points of the Sytox Green-based kinetics showed a clear increase in NET formation with increasing pH; the changes were small and slow in the negative control, whereas the increase was much higher for A2387 and ionomycin (Figures S1D–F in Supplementary Material). For example, the slope of spontaneous NET formation after 120 min was ~10-fold less steep (3.754) than that of A23187 (35.92) and ionomycin (32.92)-mediated NET formation, indicating that the effect of pH on calcium ionophore-mediated NET formation was much higher than the spontaneous NET formation. After 4 h, the cells were fixed, and fluorescence images were captured to compare the differences in the overall structure of the nuclei and NETs between the lowest and the highest pH conditions (6.6 and 7.8; Figure 1D). These images confirmed the SYTOX Green plate reader data set. Overall, pH affected both spontaneous and calcium ionophore-mediated NET formation; pH above the normal blood pH of ~7.4 promoted higher levels of NET formation, whereas a more acidic pH suppressed NET formation. The effect was very dramatic in calcium ionophore-mediated NET formation compared to spontaneous NET formation.

### Calcium Ionophores Promote Drastic Alkalinization of Neutrophils

**Figure 2:**
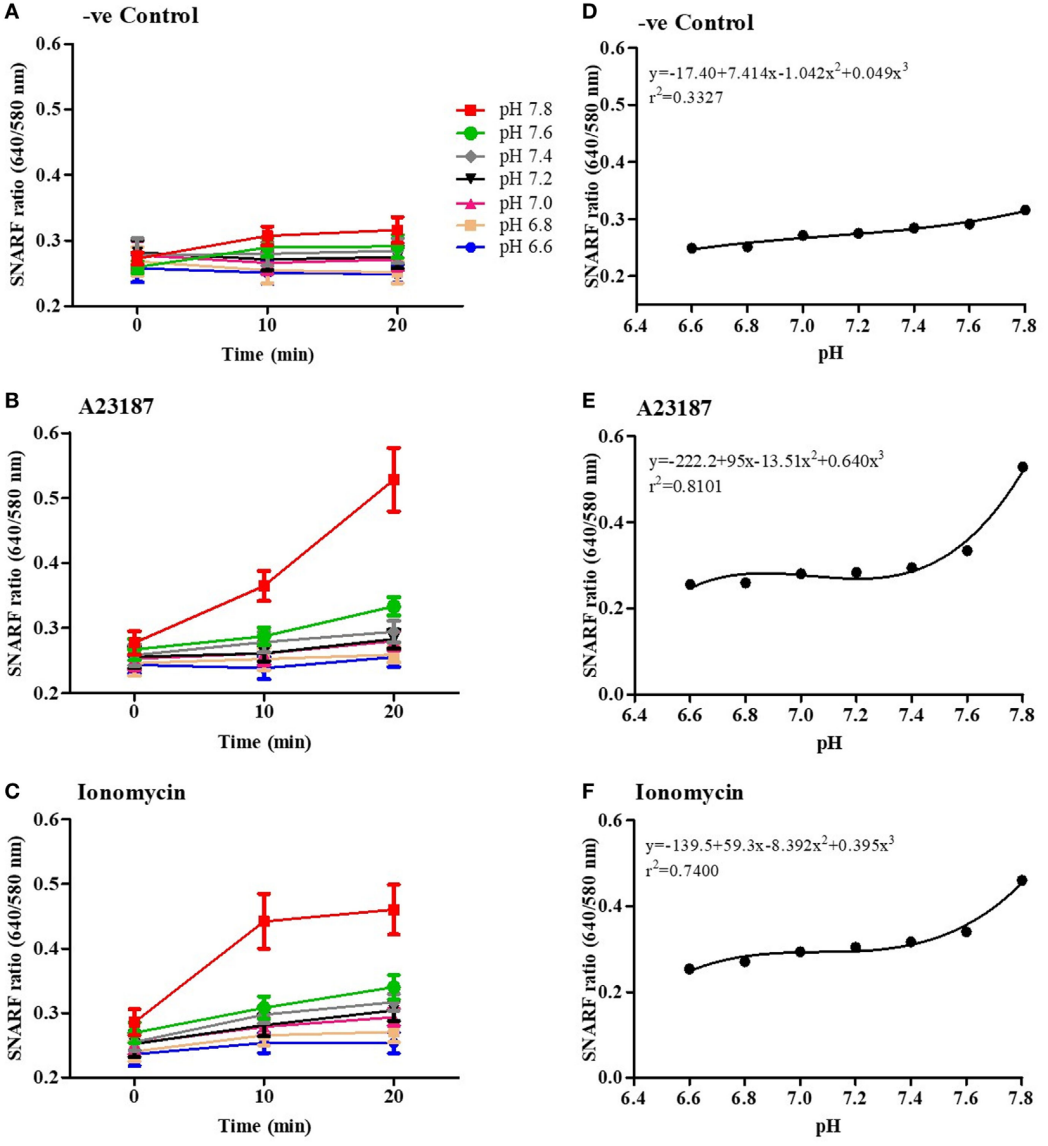
NOX-independent neutrophil extracellular trap formation stimuli drastically increase intracellular pH. Intracellular pH of the neutrophils in resting (negative control) and A23187 or ionomycin conditions was determined using the fluorescent probe SNARF. Readings were done at time point, 0, 10, and 20 min. The fluorescence kinetics analysis shows that, after 10 min, extracellular pH alters intracellular pH in negative control **(A)**, A23187 **(B)**, or ionomycin conditions **(C)**. **(D–F)** Polynomial regression for the last time point (20 min) shows that the intracellular pH values after stimulation with A23187 or ionomycin are higher than the control in higher pHs.

To determine the effect of calcium ionophores on intracellular pH (pH_i_) of neutrophils in the presence of different extracellular pH (pH_e_), we used a pH sensitive dual-wavelength Seminaphtharhodafluor (SNARF) dye. SNARF-loaded neutrophils were resuspended in media with seven different pH_e_, from 6.6 to 7.8, with or without A23187 or ionomycin and SNARF ratios were calculated at 0, 10, and 20 min time points. The 20-min time points were chosen to analyze the intracellular pH change because the majority (87–98%) of the cells were alive at that time point, excluding any bias related to extracellular buffers entering the dying cells without any cellular regulation (Table S1 in Supplementary Material). In this format, increasing SNARF ratios (ratio 640/580 nm) reflect higher intracellular pH values (Figures 2A–C). In non-stimulated neutrophils, increasing pH_e_ increased the pH_i_ and reached a slope of 7.4*x* + 0.05*x*^3^ SNARF ratios per pH unit at 20 min (Figure 2A). By contrast, at 20 min stimulation with A23187 (a slope of 95*x* + 0.64*x*^3^; Figure 2B) or ionomycin (a slope of 59.3*x* + 0.4*x*^3^; Figure 2C), much higher increase in pH_i_ values were detected compared to their respective controls (Figures 2D–F; see the full non-linear equations on the graphs). For calcium ionophore conditions, a drastic increase in pH_i_ was apparent beyond pH_e_ of 7.6. These results indicate that both of the calcium ionophores drastically increase intracellular pH at the beginning of NOX-independent NET formation process.

### Higher pH Induces Calcium Influx in Resting and A23187- or Ionomycin-Activated Neutrophils

**Figure 3:**
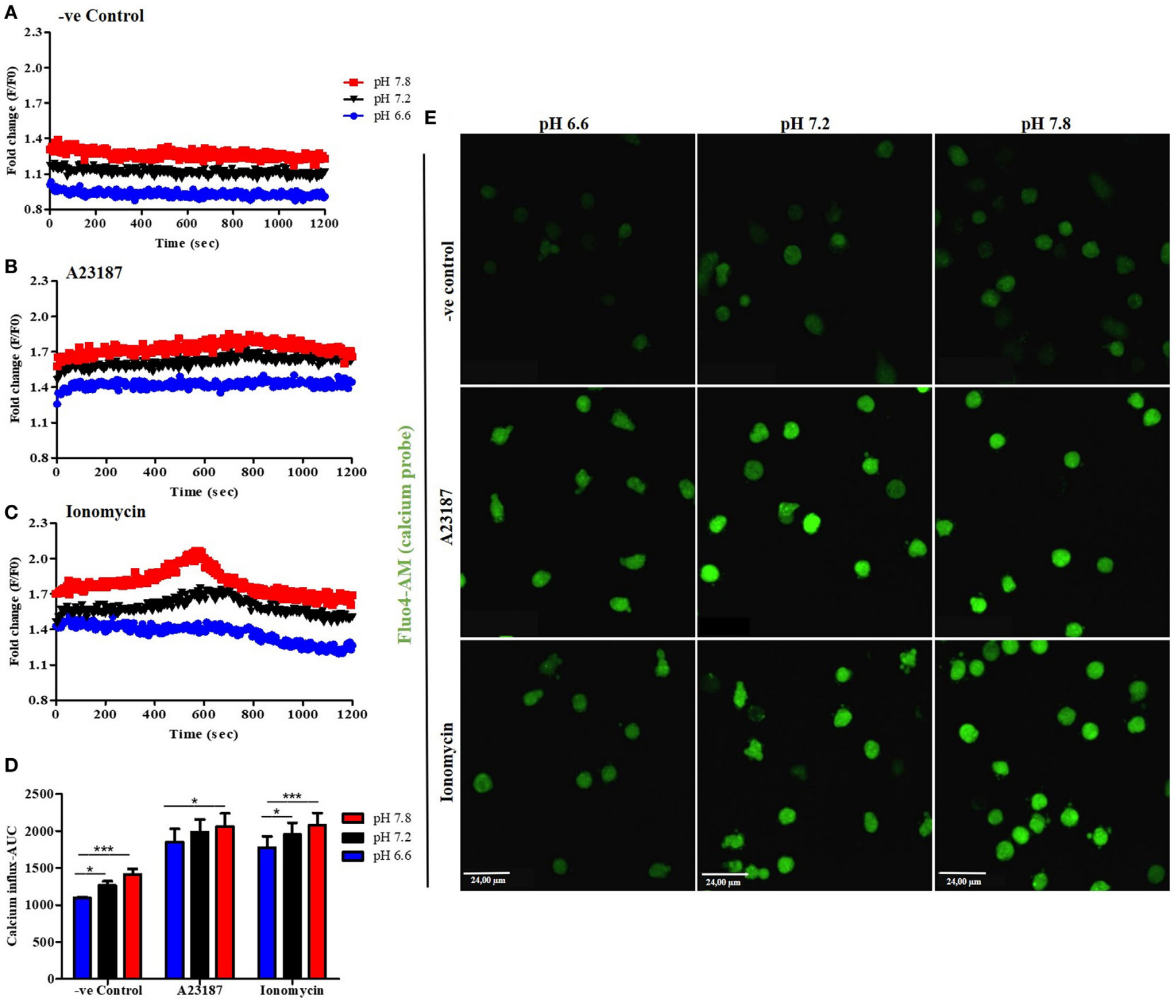
Alkalinization increases intracellular calcium influx. Neutrophils preloaded with calcium probe Fluo-4 AM were incubated for 15 min in HBSS calcium-free media. After washing, neutrophils were incubated in three different RPMI pH levels and stimulated or not with A23187 or ionomycin. Even in resting cells, at higher pHs, the calcium influx/mobilization is increased **(A)**. The same occurs after stimulation with A23187 or ionomycin, but to much higher levels **(B,C)**. Area under the curve (AUC) of three independent experiments shows difference between pH 7.2 and 7.8 compared to 6.6 in resting cells or ionomycin-treated cells. A23187 shows difference between pH 6.6 and 7.8 conditions **(D)**. Fluorescence microscopy images corroborate the plate reader assays and show an increase of calcium influx after stimulation of neutrophils at higher pH buffers **(E)**. Two-way ANOVA with Bonferroni’s post-test. *n* = 3; *p < 0.05, ***p < 0.001. See Figure S4 in Supplementary Material, which indicates that pH does not directly affect Fluo-4 AM florescence intensity.

Increase in intracellular calcium (Ca^2+^) is important for increasing NET formation-related cellular functions (5). However, the effect of pH on calcium influx in neutrophils is not clearly established. Therefore, we asked whether pH levels could affect the intracellular Ca^2+^ levels in resting and activated neutrophils. First, neutrophils were preloaded with the Ca^2+^ dye Fluo-4 AM and activated with media (negative control), A23187, or ionomycin in three different pH conditions (6.6, 7.2, and 7.8). Cytosolic Ca^2+^ concentration was measured every 30 s for 20 min. Plate reader assays showed that increased pH facilitated Ca^2+^ influx in resting and stimulated neutrophils (Figures 3A–D; increasing pH does not increase the fluorescence of Fluo-4 AM dye, Figure S3 in Supplementary Material). The magnitude of calcium increase is about the same (~0.2-fold) between two pHs; however, the total calcium levels are much higher in the presence of calcium ionophores. To confirm the effect of pH in Ca^2+^ influx, we performed fluorescence imaging, in resting (negative media) or calcium ionophore-activated neutrophils (A23187 or ionomycin), in three different pH conditions (6.6, 7.2, and 7.8; Figure 3E). Images were taken directly after cells stimulation. This data set confirmed the plate reader data showing an increase of the fluorescence in higher pH conditions. Therefore, increasing pH helps to increase intracellular calcium levels in neutrophils, particularly in the presence of calcium ionophores.

### mROS Is a Key Factor Regulating the pH Effect on NOX-Independent NETosis

**Figure 4:**
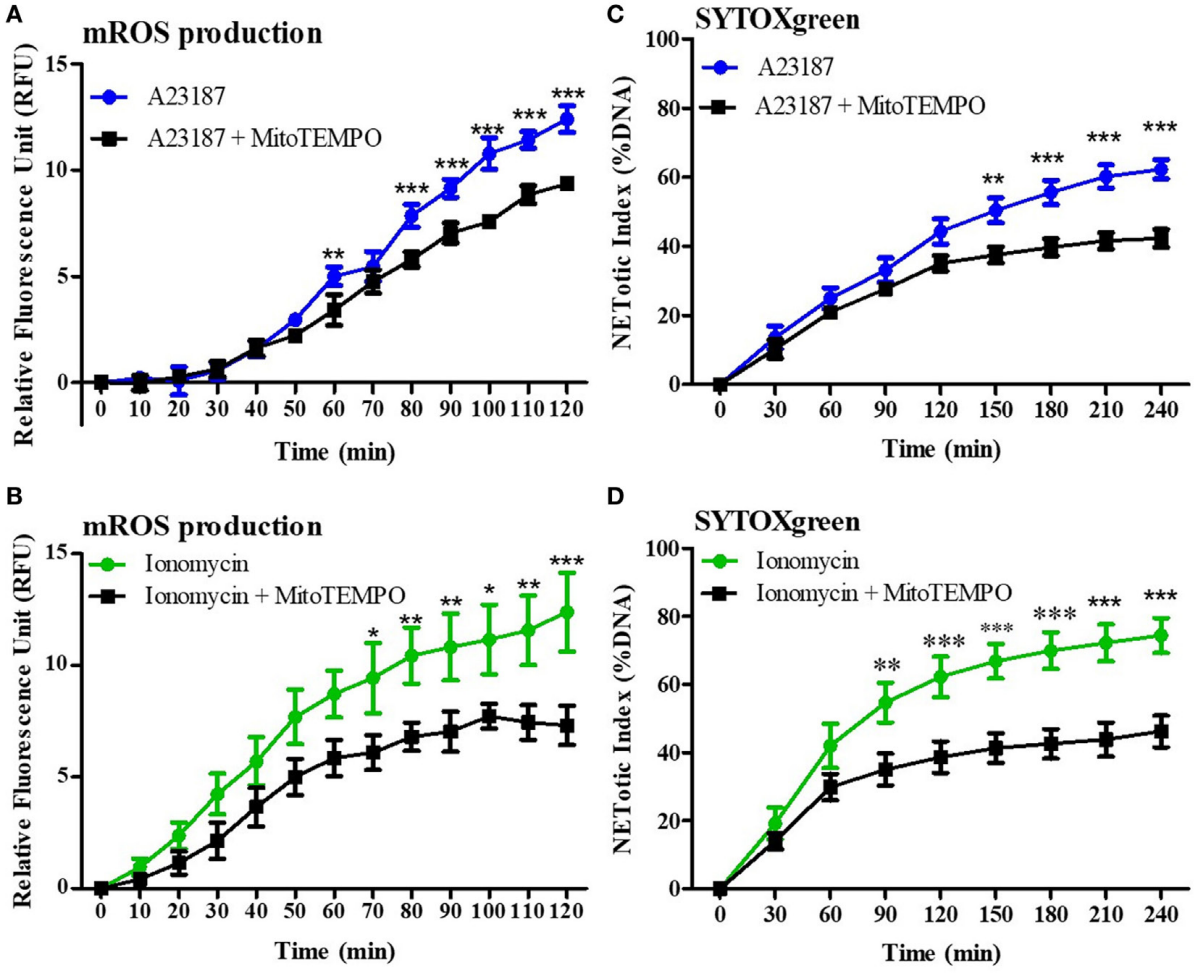
Mitochondrial reactive oxygen species (mROS) is a key factor for NOX-independent neutrophil extracellular trap (NET) formation. Purified neutrophils, at physiologic pH conditions (7.4), were incubated with or without 200 µM MitoTEMPO, an mROS scavenger, for 15 min and 4 µM of MitoSOX, a specific probe to detect mROS production. Neutrophils were then stimulated with A23187 or ionomycin, and mROS production was assessed. Readings were done every 4 min up to 120 min. MitoTEMPO decreases mROS induced by A23187 and ionomycin **(A,B)**. To evaluate the importance of mROS production for NET formation, neutrophils were incubated with or without MitoTEMPO, and a SYTOX Green assay was performed. Readings were done every 30 min up to 4 h. mROS scavenger reduces NET formation after A23187 or ionomycin stimulation **(C,D)**. *n* = 5, two-way ANOVA with Bonferroni’s post-test. *p < 0.05, **p < 0.01, ***p < 0.001. See Figure S5 in Supplementary Material, for the mROS production in NOX-dependent and NOX-independent agonists.

Mitochondrial reactive oxygen species production is related to NOX-independent NET formation (5, 14, 15). Therefore, first we verified the importance of mROS for NOX-independent NET formation by performing MitoSOX (a specific fluorescent probe for mROS detection) and SYTOX Green assays in the presence of an mROS scavenger (MitoTEMPO). Purified neutrophils were incubated with 200 µM MitoTEMPO for 15 min under physiologic pH conditions (7.4), and stimulated with media (negative control), A23187, or ionomycin. MitoTEMPO decreased mROS generation induced by both stimuli (Figures 4A,B). SYTOX Green assays showed that NET formation was also significantly decreased in the presence of mROS scavenger, confirming that mROS production is a key factor for NOX-independent NET formation (Figures 4C,D). Using PMA (a prototypical agonist for the NOX-dependent NET formation), we confirmed that mROS does not play a major role in NOX-dependent NET formation (Figure S4 in Supplementary Material).

**Figure 5:**
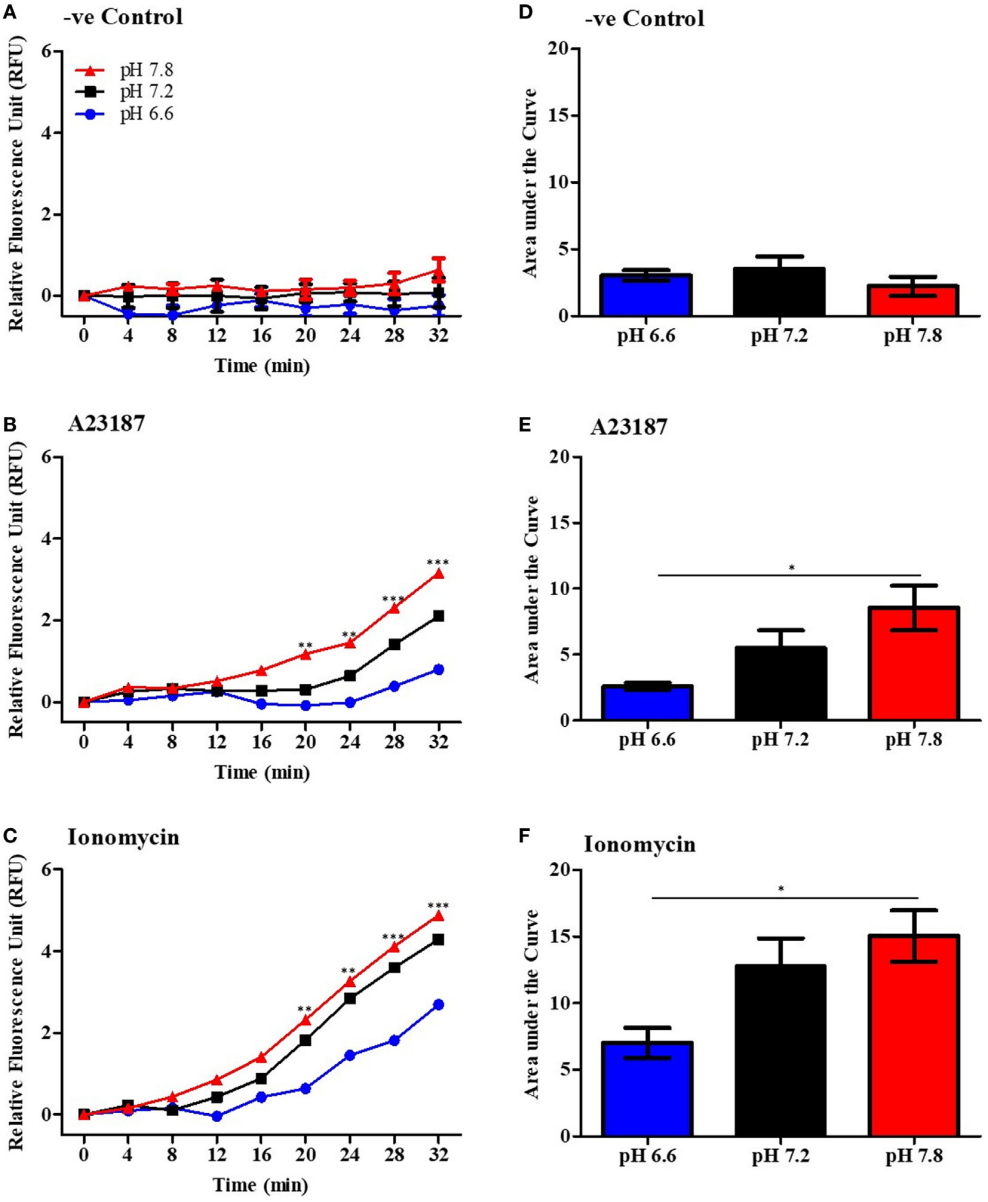
Higher pH increases mitochondrial reactive oxygen species (mROS) production by neutrophils after stimulation by A23187 and ionomycin. Purified neutrophils were resuspended in RPMI at different pHs (ranging from 6.6 to 7.8) and incubated with 4 µM of mitoSOX. Cells were seeded in a 96-well plate and stimulated with A23187 or ionomycin. The mROS production was measured every 4 min up to 32 min. Time-course of mROS production in negative control **(A)**, A23187 **(B)**, or ionomycin **(C)**. Area under curve was calculated to measure the total mROS production after 32 min stimulation in negative control **(D)**, A23187 **(E)**, or ionomycin **(F)**. *n* = 6. Two-way ANOVA with Bonferroni’s post-test and one-way ANOVA with Bonferroni’s post-test (area under curve). *p < 0.05, **p < 0.01, ***p < 0.001. See Figure S6 in Supplementary Material for 120 time point analysis, and Figures S7 and S8 in Supplementary Material for the SYTOX Green and mROS assay after MitoTEMPO treatment in different pHs.

Since calcium influx plays an important role in the mROS production (16–19), we asked whether increasing pH could affect the mROS production. To measure mROS, neutrophils were treated with 4 µM MitoSOX, and stimulated either with media control (negative control), A23187 or ionomycin in three different pH conditions (pH 6.6, 7.2, and 7.8). mROS fluorescence in these cells was measured every 4 min up to 120 min. Within 30 min, we found that neutrophils resuspended in higher pH media generated higher amount of mROS after stimulation with either A23187 or ionomycin (Figures 5A–F). Analysis of the MitoSox data after 120 min of stimulation indicates that resting neutrophils also generate mROS at higher pH, albeit lower levels than measured in calcium ionophore-stimulated neutrophils (Figure S5 in Supplementary Material). We also measured the MitoTEMPO-mediated suppression of mROS production measured by MitoSox and NET formation measured by three other pH conditions. These data suggest that increasing pH increases mROS generation and scavenging mROS suppresses NOX-independent NET formation (Figures S6 and S7 in Supplementary Material for the pHs 6.6, 7.2, and 7.8). Collectively, increasing pH increases mROS generation during the activation of neutrophils with calcium ionophores, and pH-dependent increase in mROS generation is an important component for promoting NOX-independent NET formation.

### Alkalinization Increases PAD4 Activity and Citrullination of Histone 3 during NOX-Independent NET Formation

**Figure 6:**
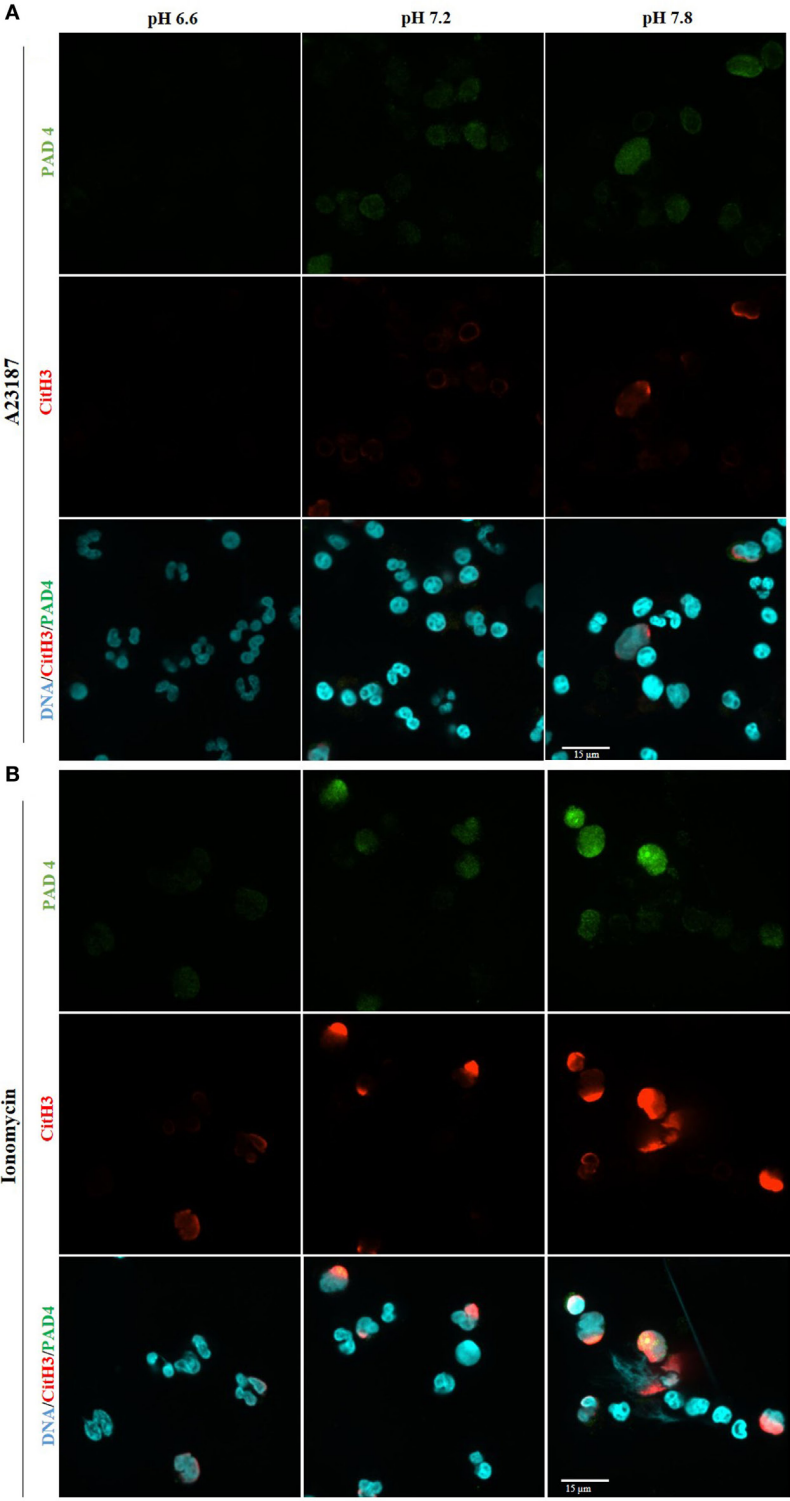
Higher pH increases PAD4 amount and activity after stimulation with A23187 or ionomycin. 1 × 10^5^ neutrophils/well were seeded in a chamber slide and stimulated in the absence or presence of A23187 or ionomycin. After incubation for 30 min, cells were fixed and immunostained for PAD4 and citrullinated histone 3, and stained for DNA. Confocal images of PAD4 and citrullinated histone 3 show an increase of PAD4 amount and activity at alkaline condition, after stimulation with A23187 **(A)** or ionomycin **(B)**. Blue, DAPI staining for DNA; green, PAD4; red, histone 3 citrullinated; *n* = 3; scale bar 22 µm. See Figure S8 in Supplementary Material, for the negative controls, and Figure S9 in Supplementary Material for the isotype controls. These images show no background staining and confirm the specificity of the antibodies.

**Figure 7:**
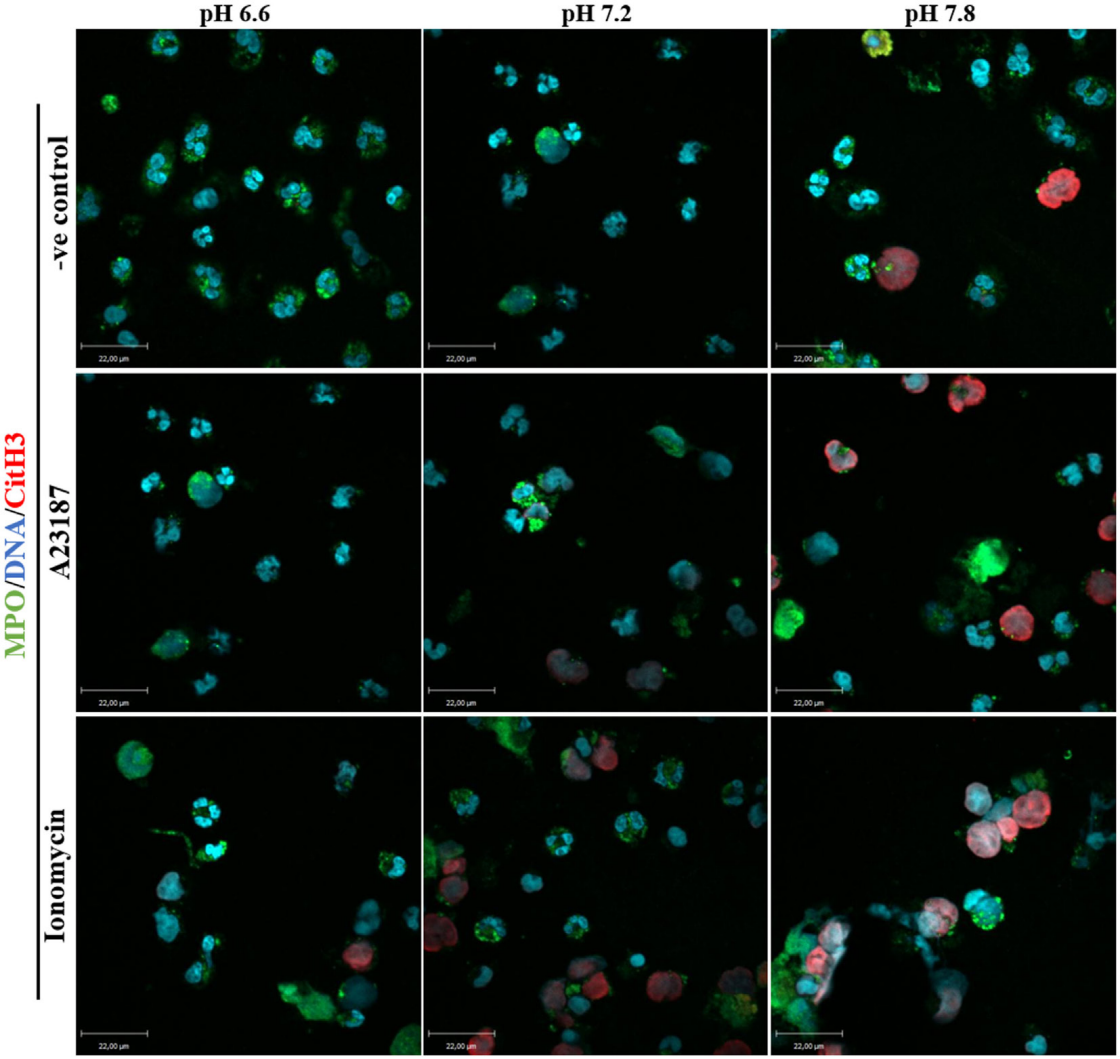
Higher pH increases citrullination of histone 3 and neutrophil extracellular trap (NET) formation in resting neutrophils and after stimulation with A23187 or ionomycin. 1 × 10^5^ neutrophils/well were seeded in a chamber slide containing 3 different pH buffers (6.6, 7.2, and 7.8). Cells in these three different pH conditions were stimulated in the absence or presence of A23187 or ionomycin. After 2 h of incubation, neutrophils were fixed and immunostained for MPO, citrullinated histone 3, and DNA. Co-localization of these markers confirms that higher pH conditions increase PAD4 activity (histone 3 citrullinated) and NET formation in control and neutrophils stimulated with A32187 or ionomycin. Blue, DAPI staining for DNA; green, MPO; red, citrullinated histone 3; *n* = 4; scale bar 22 µm. See Figures S10–S12 in Supplementary Material for the single channel images, and Figure S13 in Supplementary Material for the isotype controls. These images show no background staining and confirm the specificity of the antibodies.

PAD4 activation is a key step in NOX-independent NET formation. Once bound to cytosolic-calcium, PAD4 is activated and translocated to nucleus (6, 20, 21). The translocated PAD4 deiminates positively charged arginine present on histones 3 into non-charged citrulline, helping chromatin decondensation (22, 23). This step is especially relevant to NOX-independent NET formation (5); NET images clearly showed a strong histone 3 citrullination. To determine the influence of the pH in NET formation, we performed a confocal immunofluorescence microscopy of PAD4 and citrullinated histone 3. Purified neutrophils were seeded in a chamber slide, stimulated with buffer, A23187, or ionomycin up to 30 min, fixed and stained the cells with monoclonal antibodies to PAD4, citrullinated histone 3, and DNA dye. Neutrophils activated with A23187 or ionomycin, showed increased immunostaining for PAD4 and citrullinated histone 3 in higher pH conditions (Figures 6A,B; Figure S8 in Supplementary Material for negative controls and Figure S9 in Supplementary Material for isotype controls). Furthermore, we performed confocal microscopy of the immunostained neutrophils to determine citrullination of histone 3 in different pH conditions. Images showed a strong co-localization of MPO, DNA, and citrullination of histone 3, in neutrophils incubated in higher pH, confirming that A23187 or ionomycin-stimulated neutrophils have a greater amount of citrullination of histone 3 and formed a greater amount of NETs with increasing pH (Figure 7; Figures S10–S12 in Supplementary Material for single channels and Figure S13 in Supplementary Material for isotype control). Therefore, increasing pH increases CitH3 formation that facilitates NOX-independent NET formation.

### Histone Cleavage

**Figure 8:**
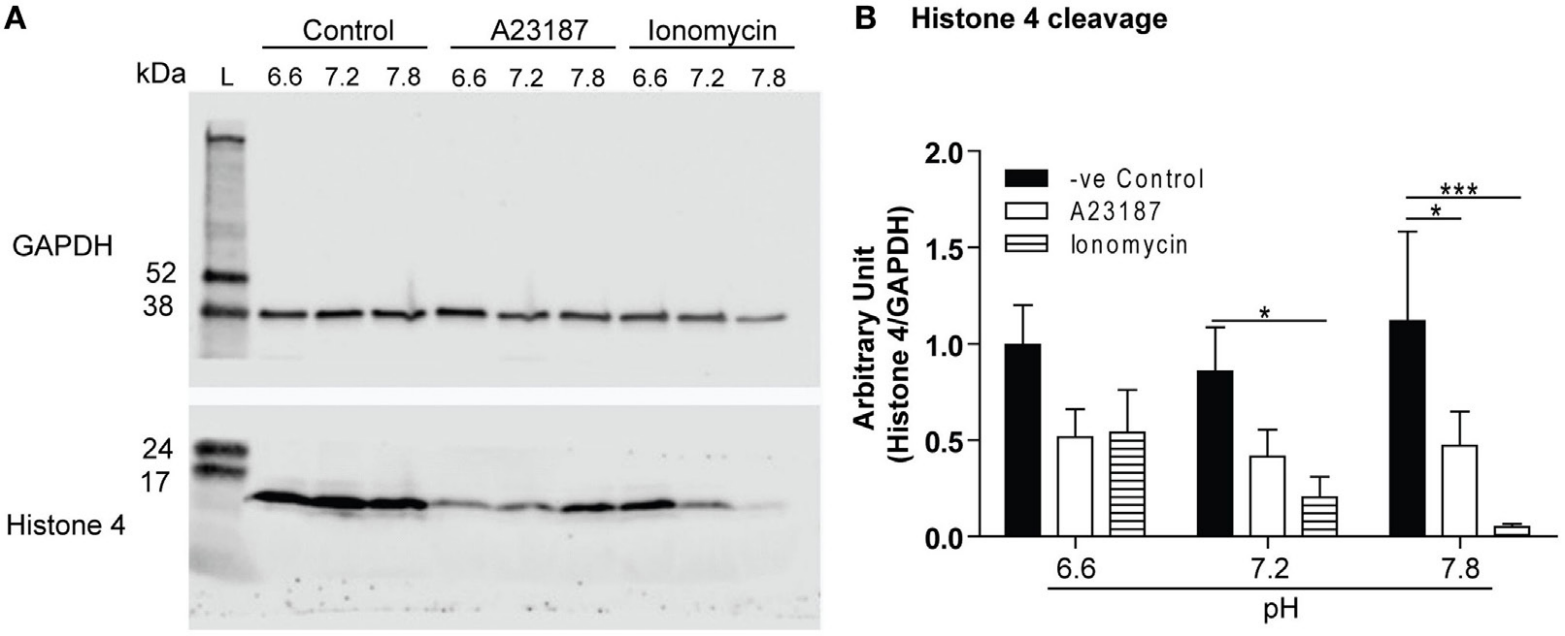
Raising pH increases Histone 4 cleavage. Histone H4 immunoblot analysis was performed by using neutrophils stimulated with either media (negative control), A23187, or ionomycin in different pHs (6.6, 7.2, or 7.8). **(A)** Histone H4 immunoblot shows histone cleavage during A23187- and ionomycin-induced neutrophil extracellular trap (NET) formation, without substantial cleavage observed in spontaneous NET formation. GAPDH blots were used as a loading controls (*n* = 7). **(B)** The densitometry data of each H4 bands were normalized with GAPDH. The densitometry data show that increase in pH promotes H4 cleavage in A23187- and ionomycin-mediated NET formation (*n* = 7; two-way ANOVA with Bonferroni’s post-test. *p < 0.05, ***p < 0.001).

Histone 4 cleavage is another hallmark event of NET formation; therefore, we sought to determine whether the pH levels affect this process. The cells were seeded in three different media conditions (pH 6.6, 7.2, or 7.8) with media (negative control) or calcium ionophores (A23187 or ionomycin), and after 1 h the H4 cleavage was determined by Western blot analysis. Unstimulated controls had little or no histone cleavage, whereas both calcium ionophores promoted substantial histone cleavage. The pH-dependent effect was much clear for ionomycin condition (Figure 8). Therefore, increased cleavage of histone is another factor that could promote NET formation at higher pHs, particularly during the activation of neutrophils by calcium ionophores.

## Discussion

Blood pH is strictly maintained at ~7.34; however, local tissue pH changes drastically, often acidifying inflamed areas (12, 24–26). During sterile inflammation in joints, for example, the synovial fluid pH drops to 6.0–7.0 (27). A recent study demonstrated that the alkaline pH of the pancreatic ducts can cause increased NET formation, which blocks pancreatic ducts and promotes pancreatitis (28). However, the mechanistic details of pH-mediated regulation of NET formation are not clearly understood. In this study, we confirm that alkaline pH increases calcium ionophore A23187- and ionomycin-induced NOX-independent NET formation, and provide several mechanistic details. We identified that increase in extracellular pH promotes intracellular calcium concentration, mROS generation, histone citrullination, and histone cleavage. This effect is dramatic in the presence of calcium ionophores such as A23187 or ionomycin, a compound secreted by antibiotics producing *Streptomyces*, a group of Gram-positive bacteria. These findings provide better understanding of the mechanism as to how pH regulates calcium-mediated NOX-independent NET formation.

Our study shows that alkaline pH increases NET formation, whereas acidic pH suppresses NET formation (Figure 1). This is consistent with studies published while our manuscript was in preparation (12, 13). We demonstrated that neutrophils equilibrate their pHi with the pHe within minutes, and a dramatic pHi increase occurs after neutrophil stimulation with NOX-independent NET formation agonists (Figures 2A–F). Several neutrophil functions are suppressed in acidic microenvironment, including chemotaxis, microbicidal activity, and apoptosis (11, 24, 29–33). This study further shows that an acidic environment suppresses yet another key neutrophil function, NET formation.

By contrast, some important NET formation-related enzymes and ion channels are more active in alkaline pH (11, 34). Corroborating these points, alkaline pH increases Ca^2+^ influx in resting and calcium ionophore-stimulated neutrophils (Figures 3A–E). Binding of calcium to A23187 and ionomycin increases with increasing pH (35). A23187 binds effectively to calcium at lower pH than ionomycin, and ionomycin is a more effective binder of calcium than A23187, particularly at higher pHs (35). Hence, the increase in calcium concentration during ionophore-mediated uptake at higher pHs is at least partly due to the increased binding of calcium to the ionophores. Ionomycin is secreted by antibiotics producing unusual group of filamentous Gram-positive bacteria *Streptomyces*, particularly by *S. conglobatus*. Hence, ionomycin-induced NET formation could also be relevant to issues related to bacterial defense vs. host defense mechanism.

A recent study by Maueröder and colleagues suggested that raising pH_e_ by bicarbonate increases Ca^2+^ influx in resting neutrophils (13). The data obtained in this study show an increase of intracellular Ca^2+^ influx, in both resting and activated neutrophils placed in different pH buffers that were adjusted using HCl or NaOH (Figures 2 and 3). These differences may be due to the differences in neutrophil purification and experimental media conditions. However, the overall message is that increasing pH increases Ca^2+^ influx in neutrophils.

Calcium is a crucial second messenger for many cellular functions, including mitochondrial function (36–40). In 2015, our group demonstrated that NOX-independent NET formation stimulates mROS production, suggesting that mROS generation was an important step during NOX-independent NET formation (5). Therefore, by using a specific mROS scavenger we first confirmed that mROS is important for calcium ionophore-mediated NET formation (Figure 4). Our results further show that neutrophils present in more alkaline media produce higher amount of mROS in resting and activated (e.g., A23187 or ionomycin conditions) neutrophils (Figure 5). Although MitoSox readings continued to increase until the end of the experiments, we used early time points (e.g., 20–30 min) to draw conclusions. At this time point, most of the cells were still viable. Longer time points are still relevant to non-stimulated cells that take a long time to generate mROS and to undergo cell death. The increase in mROS is much higher in activated neutrophils. MitoSox data at later time points for calcium ionophore-mediated NET formation are confounded by the dye binding to intracellular components of dying cells. However, MitoTEMPO that scavenges mROS continued to suppress MitoSox and SYTOX Green fluorescence (NET formation). Therefore, these results indicate that mROS is a key component of NOX-independent NET formation and contributes to the pH-dependent increase in NET formation induced by calcium ionnophores such as A23187 and ionomycin.

Calcium is also essential for PAD4 activation, where five Ca^2+^ molecules can bind on each PAD4 molecule and change the conformation of the enzyme (8, 21, 22, 41, 42). PAD4 is a constitutively expressed enzyme in neutrophils, and present in the cytosol (6). When PAD4 is complexed with Ca^2+^, it is able to reach the nucleus and citrullinate histones (20). This process converts positively charged arginine residues to neutral citrulline, which helps to decondense chromatin (6, 22, 23, 41). There are numerous reports describing the importance of PAD4 in citrullination of histone 3 for NET formation (43–45), and one report showed an increase of histone 3 citrullination during alkaline pHs (13). A few studies showed an increase of PAD4 after the stimulation of neutrophils with other agonists (43, 46). Immunocytochemistry images indicate that PAD4 amount and activity (determined by CitH3) increase upon activating neutrophils with NOX-independent NET formation agonists A23187 or ionomycin, at higher pH conditions (Figures 6A,B and 7). The optimal pH for PAD4 activity is 7.6–8.0 (8). Therefore, increased level and activity of PAD4 at alkaline pH would facilitate the chromatin decondensation necessary for NET formation.

Another important enzyme that participates in NET formation is neutrophils elastase (NE). It cleaves histone 4 (9, 47); histone cleavage has been linked to chromatin decondensation, and considered as an important step in NET formation (9, 47, 48). In resting neutrophils, changes in pH (pH 6.6, 7.2, and 7.8) does not significantly alter histone 4 cleavage. By contrast, increase in pH significantly increases histone 4 cleavage during the stimulation of neutrophils with calcium ionophores (Figure 8). This result corroborates with the SYTOX Green assay (Figure 1), where very low amount of NET formation was seen at pH 6.6 whereas high amount of NET formation was observed in pH 7.8. Paradoxically, the optimal pH for NE is basic (pH 7.5–8.5), whereas the optimal pH for MPO is acidic (pH 4.7–6.0) (10, 49, 50). Consistent with the optimal pH of NE (10), higher histone 4 cleavage was detected in neutrophils at alkaline than acidic pHs (Figure 8). Therefore, neutrophil proteases, but not MPO, may be important for NET formation at alkaline pH. Studies showed the importance of NE and MPO in NOX-dependent NET formation (9, 47). A recent study showed that A23187-stimulated NET formation does not require the activity of MPO enzyme (51). Therefore, NE and MPO activities are necessary to drive the NOX-dependent NET formation, but the neutrophil proteases may be more important for NOX-independent NET formation.

What type of NET formation occurs during calcium ionophore-mediated NET formation and other forms of NETosis is not fully resolved (52). In general, suicidal NETosis could be either Nox dependent or Nox independent, but both use nuclear DNA and the cells “almost die” at the end of the process. Some reports suggest that neutrophils with no nuclear DNA could survive for some more time as cytoplasts (53–55). By contrast, vital NET formation involves Nox-independent nuclear DNA bleb release (3) and mitochondrial DNA release (56). This form of vital NETosis is considered to be very rapid (5–15 min), and neutrophils continued to survive (57). Subsequent work established that agonists such as calcium ionophores A23187 and ionomycin stimulate Nox-independent NET formation that leads to suicidal NETosis (4, 5, 14, 58–61). We have previously characterized several mechanistic steps involved in calcium-induced Nox-independent suicidal NETosis that requires mROS (5). Nox inhibitor DPI does not inhibit calcium ionophore-mediated NETosis (5). Therefore, suicidal NET formation always does not require nicotinamide adenine dinucleotide phosphate oxidase-mediated oxidative burst. The data presented in this paper are related to calcium-dependent Nox-independent suicidal NET formation.

In terms of pathobiological significance, the findings presented in this paper are likely to be relevant to an inflammatory microenvironment that is often characterized by acidosis. It has been suggested that neutrophils can sense the pH changes between the border (neutral or alkaline conditions) and the center of the inflamed area (acidic conditions) (13). This type of pH sensing could help to regulate NET formation at the center to avoid excessive tissue damage, while allowing NET formation at the border of the wounds to help reduce the spread of infection. Airways of cystic fibrosis patients are chronically inflamed and have large numbers of neutrophils (62, 63). These airways are often acidic and show defects in bacterial clearance (62, 64–67). Therefore, altering pH may be useful for treating wounds and NET-related disease conditions such as cystic fibrosis.

## Ethics Statement

The study protocol for using human blood samples was approved by the ethics committee of The Hospital for Sick Children, Toronto. All the procedures including healthy human volunteer recruitment for blood donation were performed in accordance with the ethics committee guidelines. All the volunteers participating in this study gave their signed consent prior to the blood donation.

## Author Contributions

CS and LB contributed equally to this work, conducting experiments, interpreting the data, and preparing the figures and manuscript; MK planned experiments, interpreted the data, and edited the manuscript; NC is CS’s Ph.D. supervisor and edited the manuscript. SA is LB’s Ph.D. supervisor and edited the manuscript. NS edited the manuscript. NP is the principal investigator, conceived the idea, planned experiments, supervised the study, interpreted the data, and prepared and edited the manuscript.

## Conflict of Interest Statement

The authors declare that the research was conducted in the absence of any commercial or financial relationships that could be construed as a potential conflict of interest.
