# Supplementary material for: Alkaline pH Promotes NADPH Oxidase-Independent Neutrophil Extracellular Trap Formation: A Matter of Mitochondrial Reactive Oxygen Species Generation and Citrullination and Cleavage of Histone
Source: Front Immunol. 2018 Jan 9;8:1849. doi: 10.3389/fimmu.2017.01849 (PMC5767187; doi:10.3389/fimmu.2017.01849)
Supplement: Supplementary file 1 [file Image_1.PDF]

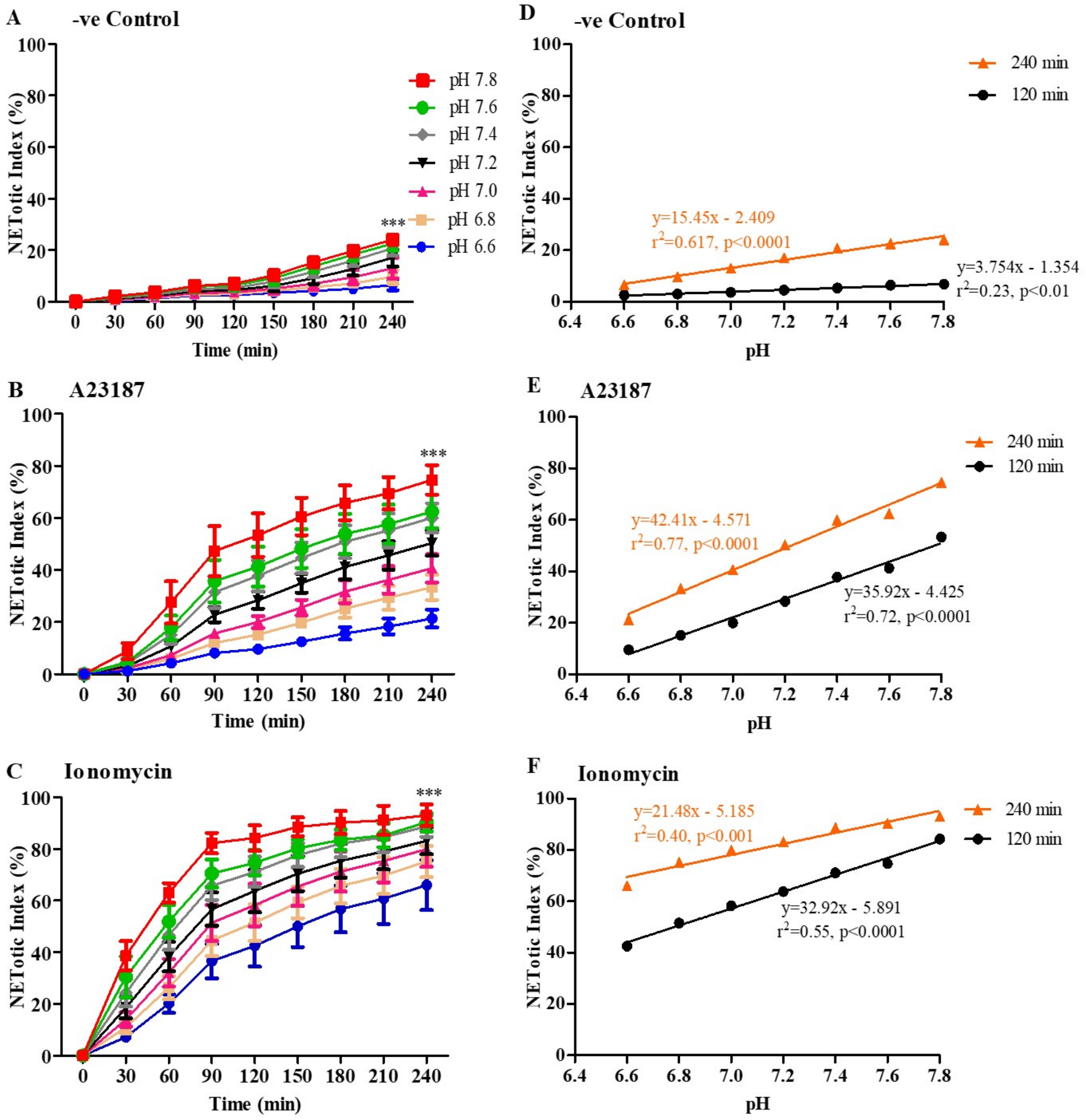

Figure S1. Raising pH increases NET formation in resting neutrophils and A23187 or ionomycin-treated neutrophils. Cells were resuspended in media with different extracellular pH buffers (6.6, 6.8, 7.0, 7.2, 7.4, 7.6 and 7.8), containing 5 µM Styox Green dye, and activated by A23187 or ionomycin or media. **(A-C)** Florescence was recorded by a plate reader for every 30 min up to 4 hours. % DNA (NETosis) released shows that neutrophils release more NETs under alkaline condition (pH 7.8), in control albeit to a low level (A; note the Y-axis scale) and after stimulation with A23187 (B) or ionomycin (C) to a much higher level. **(D-F)** The regression analyses of all seven pH conditions at 120 and 240-minute time points showed a linear increase of NETs formation with increasing pH in resting neutrophils (D), and much higher NETs release after stimulation with A23187 (E) or ionomycin (F). n=5, Two-way ANOVA with Bonferroni's post-test. ***p<0.001.
