# Supplementary material for: Alkaline pH Promotes NADPH Oxidase-Independent Neutrophil Extracellular Trap Formation: A Matter of Mitochondrial Reactive Oxygen Species Generation and Citrullination and Cleavage of Histone
Source: Front Immunol. 2018 Jan 9;8:1849. doi: 10.3389/fimmu.2017.01849 (PMC5767187; doi:10.3389/fimmu.2017.01849)
Supplement: Supplementary file 2 [file Image_2.PDF]

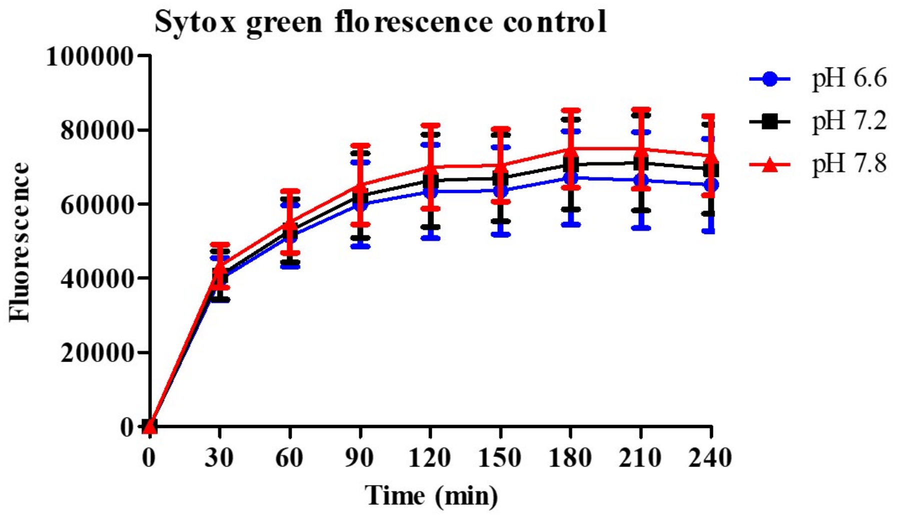

Figure S2. pH does not interfere with florescence intensity of Sytox Green dye. Same number of neutrophils, $5x10^5$, were resuspended in media with different extracellular pH buffers (6.6, 7.2 and 7.8), containing 5 µM Styox Green dye and seeded in a 96 well plate. After lysis of the cells with 10% Triton-X (1:10), florescence was recorded by a plate reader for every 30 min up to 4 hours. To show that the pH does not interfere with the florescence of Sytox Green dye, the raw numbers were obtained, analyzed and plotted after the readings. n=3, Two-way ANOVA with Bonferroni's post-test.
