# Supplementary material for: Alkaline pH Promotes NADPH Oxidase-Independent Neutrophil Extracellular Trap Formation: A Matter of Mitochondrial Reactive Oxygen Species Generation and Citrullination and Cleavage of Histone
Source: Front Immunol. 2018 Jan 9;8:1849. doi: 10.3389/fimmu.2017.01849 (PMC5767187; doi:10.3389/fimmu.2017.01849)
Supplement: Supplementary file 3 [file Image_3.PDF]

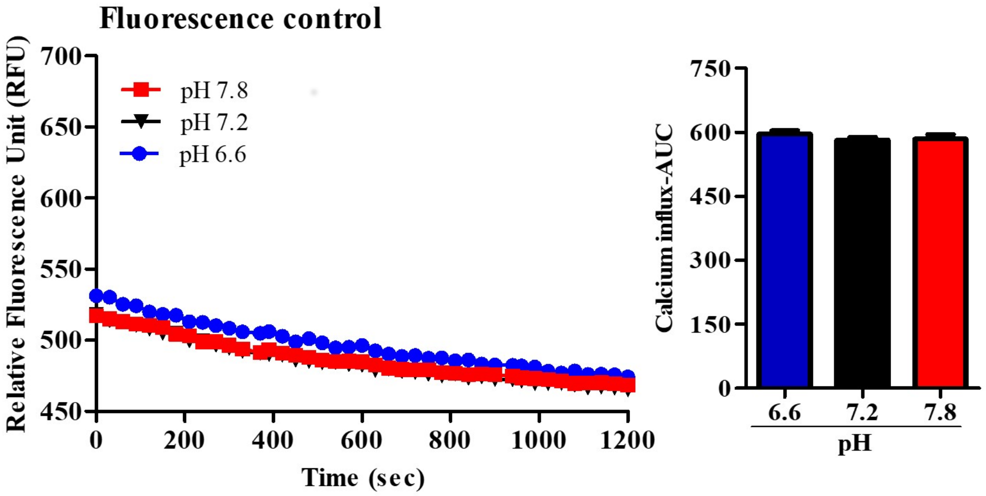

Figure S3. pH does not interfere with florescence intensity of Fluo-4 AM probe. Calcium probe was treated according to manufactures' instruction and incubated with different pH media (6.6, 7.2 or 7.8). The florescence was recorded by a plate reader every 10 seconds up to 20 minutes. The raw numbers were obtained, analyzed and plotted after the readings. A representative tracing is shown. Area under the curve (AUC) of 3 independent experiments, shows no differences among the pHs, confirming that pH does not interfere with Fluo-4 AM florescence intensity. n=3, Two-way ANOVA with Bonferroni's post-test.
