# Supplementary material for: Alkaline pH Promotes NADPH Oxidase-Independent Neutrophil Extracellular Trap Formation: A Matter of Mitochondrial Reactive Oxygen Species Generation and Citrullination and Cleavage of Histone
Source: Front Immunol. 2018 Jan 9;8:1849. doi: 10.3389/fimmu.2017.01849 (PMC5767187; doi:10.3389/fimmu.2017.01849)
Supplement: Supplementary file 4 [file Image_4.PDF]

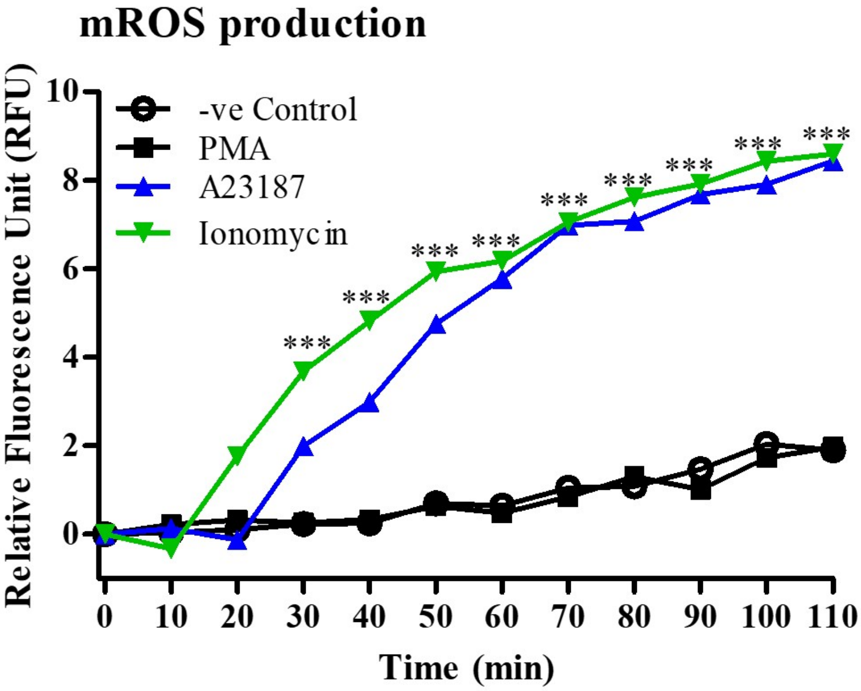

Figure S4. Calcium ionophores (A23187 or ionomycin) but not PMA induce mROS production. Purified neutrophils (1x10^5), at physiologic pH conditions (7.4), were incubated with 4 µM mROS fluorescent probe (MitoSOX) and stimulated with media control, A23187, ionomycin and PMA (NOX-dependent NETs formation stimulus). mROS production was detected only after stimulating neutrophils with NOX-independent agonists (e.g., A23187 or ionomycin) but not after NOX-dependent agonist (e.g. PMA).
