# Supplementary material for: Alkaline pH Promotes NADPH Oxidase-Independent Neutrophil Extracellular Trap Formation: A Matter of Mitochondrial Reactive Oxygen Species Generation and Citrullination and Cleavage of Histone
Source: Front Immunol. 2018 Jan 9;8:1849. doi: 10.3389/fimmu.2017.01849 (PMC5767187; doi:10.3389/fimmu.2017.01849)
Supplement: Supplementary file 5 [file Image_5.PDF]

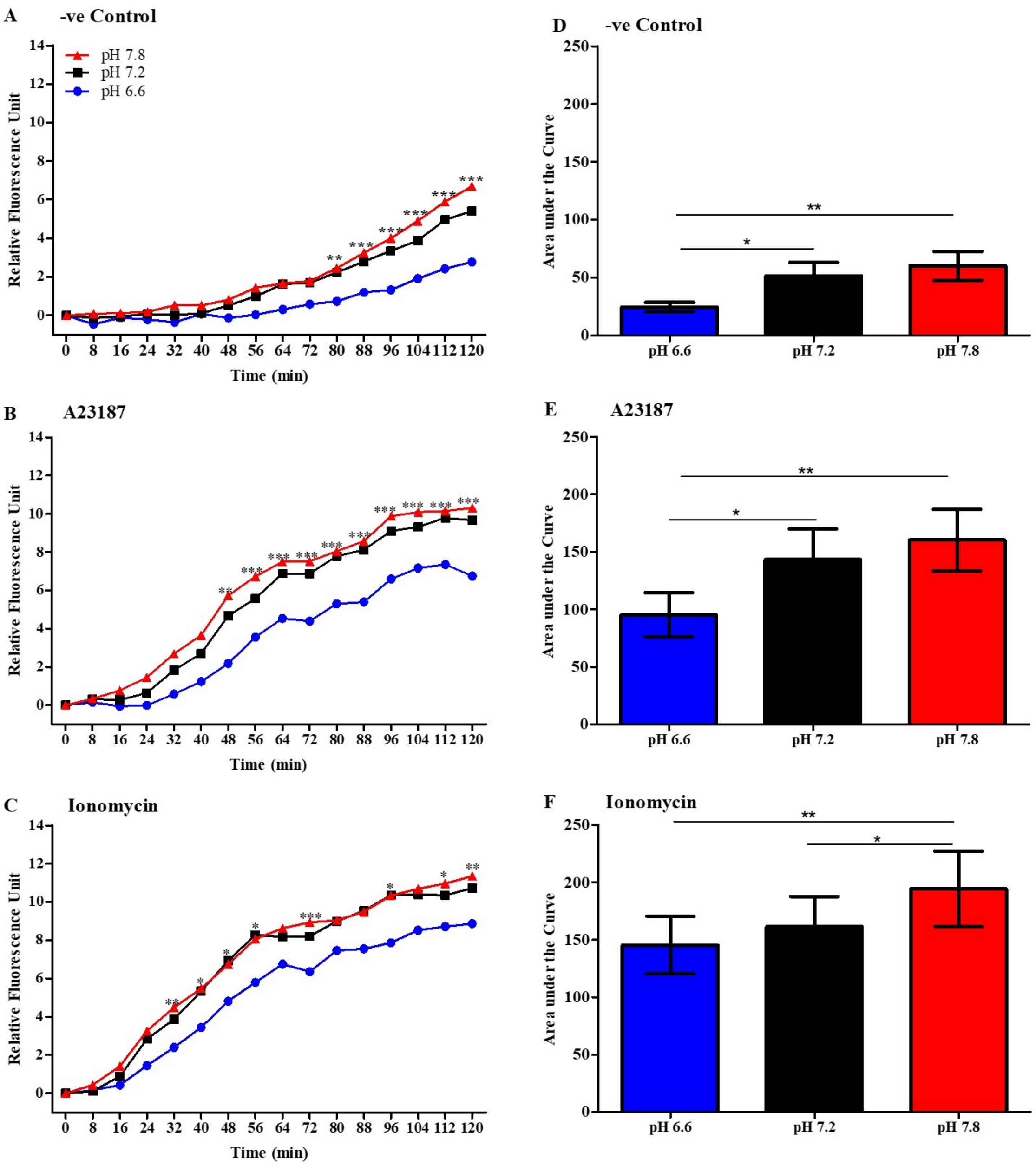

Figure S5. Higher pH increases mROS production by neutrophils. Purified neutrophils ($1x10^5$) were resuspended in RPMI in different pHs (ranging from 6.6 to 7.8) and incubated with 4 µM of MitoSOX. Cells were seeded in a 96 wells plate and stimulated with A23187 or Ionomycin. The mROS production was measured every 8 min up to 120 min. Time-course of mROS production in -ve control (A), A23187 (B) or ionomycin (C). Area under curve was calculated to measure the total mROS production after 120 minutes stimulation in -ve control (D), A23187 (E) or ionomycin (F). n= 6. Two-way ANOVA with Bonferroni's post-test and One-way ANOVA with Bonferroni's post-test (Area under curve). *p<0.05, **p< 0.01, ***p<0.001. Note: For A23187 and ionomycin conditions, differences are reliable up to ~30 min because the cell viability (lysis) increases after that time point.
