# Supplementary material for: Alkaline pH Promotes NADPH Oxidase-Independent Neutrophil Extracellular Trap Formation: A Matter of Mitochondrial Reactive Oxygen Species Generation and Citrullination and Cleavage of Histone
Source: Front Immunol. 2018 Jan 9;8:1849. doi: 10.3389/fimmu.2017.01849 (PMC5767187; doi:10.3389/fimmu.2017.01849)
Supplement: Supplementary file 6 [file Image_6.PDF]

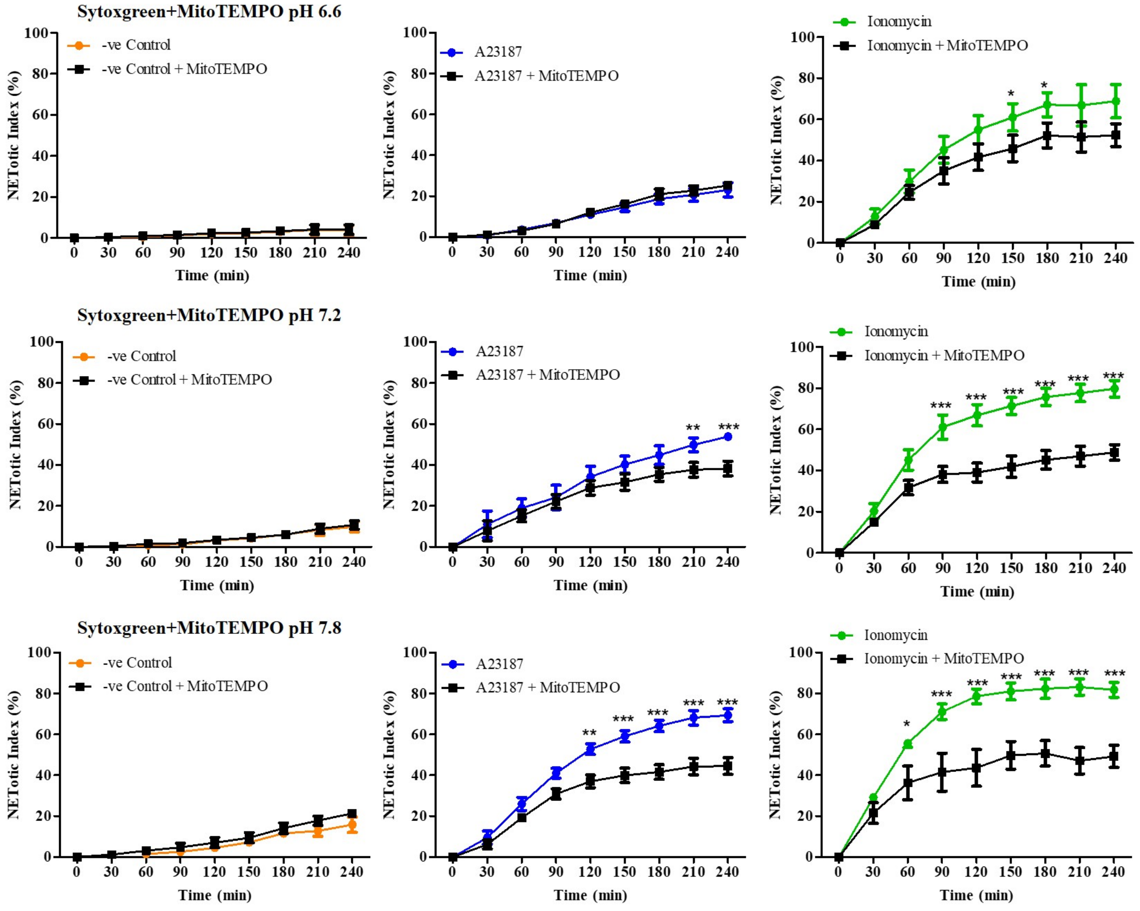

Figure S6. NETs production inhibition by MitoTEMPO in different pHs. Purified neutrophils were resuspended in RPMI in different pHs (ranging from 6.6 to 7.8) and incubated with 5 µM of SYTOX green and 200 µM MitoTEMPO. Cells were seeded in a 96 wells plate and stimulated with media (-ve control), A23187 or Ionomycin. Florescence was recorded by a plate reader every 30 min up to 4 hours. % DNA release (NETotic index) shows higher inhibition by MitoTEMPO in pH 7.8 (bottom panel) compared to pH 6.6 (top panel). n= 4. Two-way ANOVA with Bonferroni's post-test and One-way ANOVA with Bonferroni's post-test. *$p<0.05$, **$p< 0.01$, ***$p<0.001$.
