# Supplementary material for: Alkaline pH Promotes NADPH Oxidase-Independent Neutrophil Extracellular Trap Formation: A Matter of Mitochondrial Reactive Oxygen Species Generation and Citrullination and Cleavage of Histone
Source: Front Immunol. 2018 Jan 9;8:1849. doi: 10.3389/fimmu.2017.01849 (PMC5767187; doi:10.3389/fimmu.2017.01849)
Supplement: Supplementary file 7 [file Image_7.PDF]

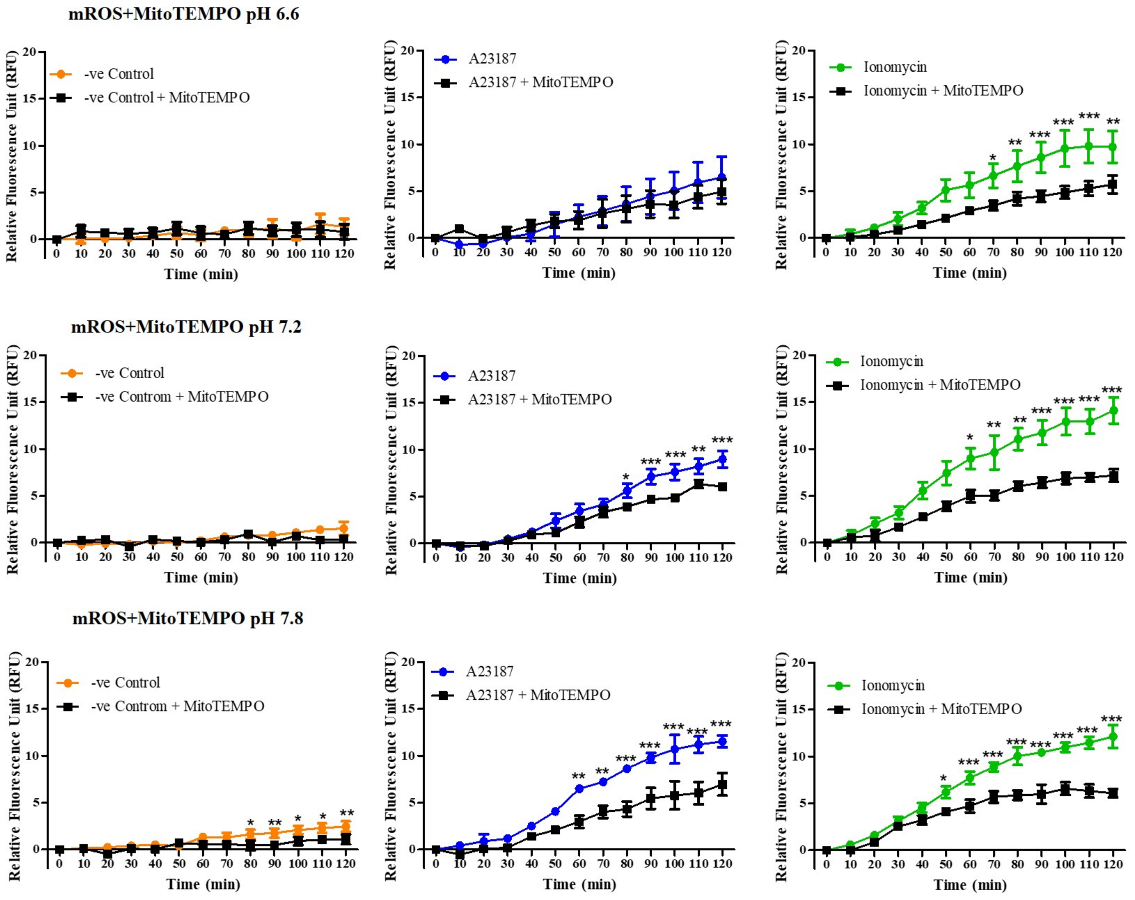

ure S7. mROS production inhibition by MitoTEMPO in different pHs. Purified neutrophils ($1x10^5$) were resuspended in RPMI in different pHs (ranging from 6.6 to 7.8) and incubated with 4 µM of mitoSOX and 200 µM MitoTEMPO. Cells were seeded in a 96 wells plate and stimulated with A23187 or Ionomycin. The mROS production was measured every 10 min up to 120 min. Time-course of mROS production in -ve control, A23187 or ionomycin in pH 6.6 (top panel), pH 7.2 (middle panel) or pH 7.8 (bottom panel). n= 4. Two-way ANOVA with Bonferroni's post-test and One-way ANOVA with Bonferroni's post-test. *$p<0.05$, **$p< 0.01$, ***$p<0.001$.
