# Supplementary material for: Alkaline pH Promotes NADPH Oxidase-Independent Neutrophil Extracellular Trap Formation: A Matter of Mitochondrial Reactive Oxygen Species Generation and Citrullination and Cleavage of Histone
Source: Front Immunol. 2018 Jan 9;8:1849. doi: 10.3389/fimmu.2017.01849 (PMC5767187; doi:10.3389/fimmu.2017.01849)
Supplement: Supplementary file 8 [file Image_8.PDF]

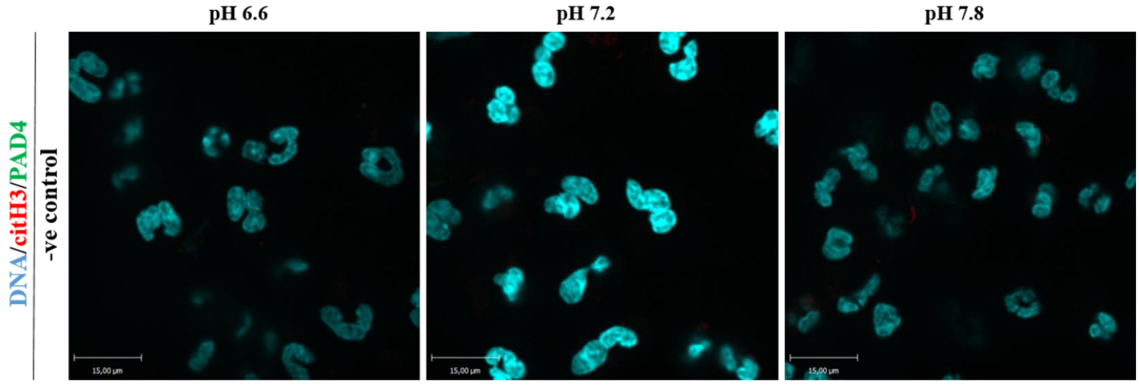

Figure S8. Higher pH does not interfere with PAD4 amount and activity in resting neutrophils after 30 minutes. $1x10^5$ neutrophils/well were seeded in a chamber slide and after incubation for 30 min with different pHs (6.6, 7.2 or 7.8), cells were fixed and immunostained for PAD4, citrullinated histone 3 and DNA. Confocal images of PAD4 and citrullinated histone 3 show that no PAD4 activity is detectable in resting neutrophils at any pH conditions. Blue, DAPI staining for DNA; Green, PAD4; Red, citrullinated histone 3; n = 3; scale bar 22 µm.
