# Supplementary material for: Alkaline pH Promotes NADPH Oxidase-Independent Neutrophil Extracellular Trap Formation: A Matter of Mitochondrial Reactive Oxygen Species Generation and Citrullination and Cleavage of Histone
Source: Front Immunol. 2018 Jan 9;8:1849. doi: 10.3389/fimmu.2017.01849 (PMC5767187; doi:10.3389/fimmu.2017.01849)
Supplement: Supplementary file 9 [file Image_9.PDF]

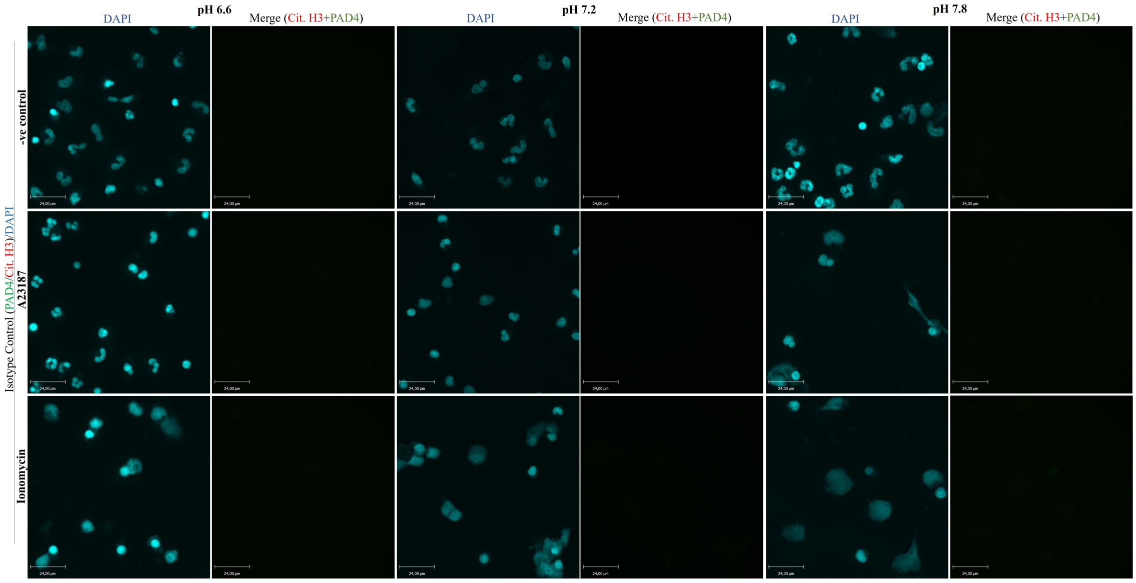

Figure S9. Isotype controls for PAD4 immunostaining. Cells treatments were performed as described in Figure 6. However, the neutrophils were incubated with mouse IgG-Thermo Fisher- (instead of PAD4) and rabbit IgG (instead of citH3) isotype controls as primary antibodies. After 1h incubation followed by 2 washes with PBS 1x the cells were incubated with secondary antibodies and DAPI. Blue, DAPI staining for DNA; Green, mouse IgG isotype control (to PAD4); Red, rabbit IgG isotype control (to citrullinated histone 3)- Thermo Fisher; scale bar 49 µm.
