# Supplementary material for: Alkaline pH Promotes NADPH Oxidase-Independent Neutrophil Extracellular Trap Formation: A Matter of Mitochondrial Reactive Oxygen Species Generation and Citrullination and Cleavage of Histone
Source: Front Immunol. 2018 Jan 9;8:1849. doi: 10.3389/fimmu.2017.01849 (PMC5767187; doi:10.3389/fimmu.2017.01849)
Supplement: Supplementary file 12 [file Image_12.PDF]

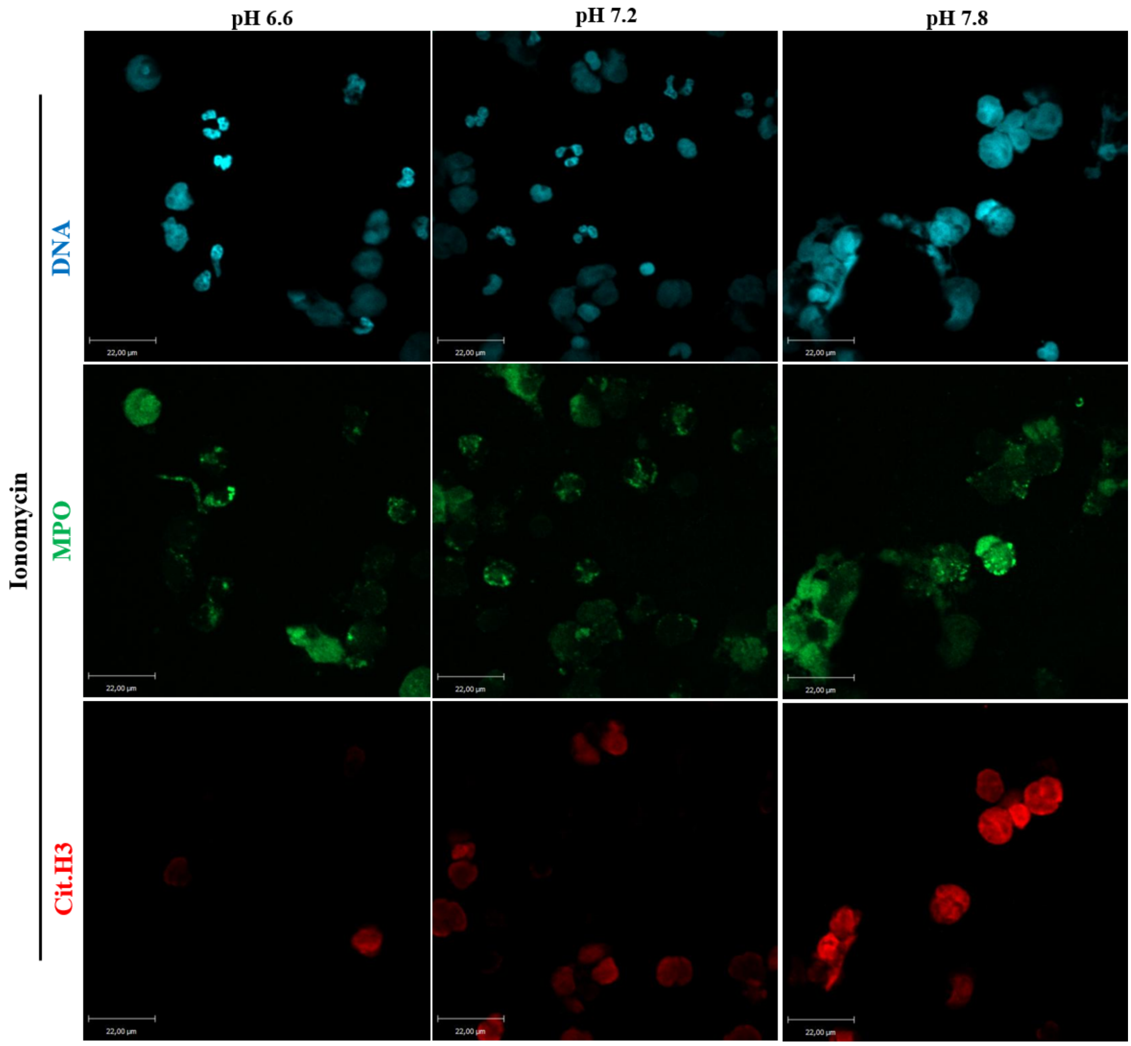

Figure S12. Single channel images showing that higher pH increases citrullination of histone 3 and NETs formation in resting neutrophils. Cells treatment were performed as described in Figure 7 (2 hours incubation). Blue=DAPI staining for DNA; Green=MPO; Red=citrullinated histone 3 in control (Fig. S10), A23187 (Fig. S11) and Ionomycin (Fig. S12) treated cells. n=4; scale bar 22 µm.
