# Supplementary material for: Alkaline pH Promotes NADPH Oxidase-Independent Neutrophil Extracellular Trap Formation: A Matter of Mitochondrial Reactive Oxygen Species Generation and Citrullination and Cleavage of Histone
Source: Front Immunol. 2018 Jan 9;8:1849. doi: 10.3389/fimmu.2017.01849 (PMC5767187; doi:10.3389/fimmu.2017.01849)
Supplement: Supplementary file 13 [file Image_13.PDF]

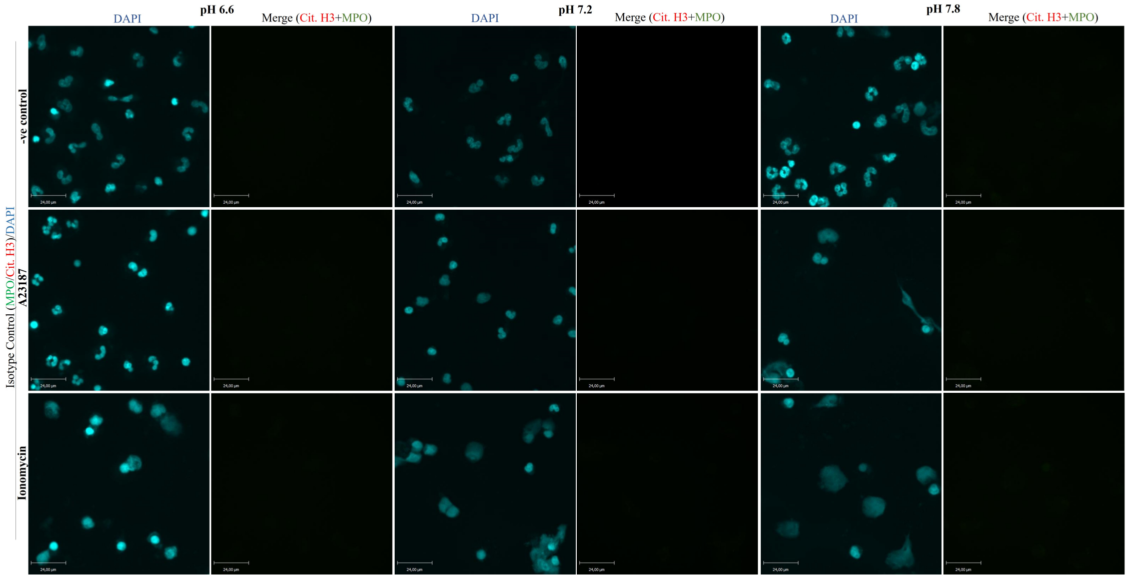

Figure S13. Isotype controls for MPO and citH3 immunostaining. Neutrophils were treated as described in Figure 7. However, cells were incubated with mouse IgG-Thermo Fisher®- (instead of MPO) and rabbit IgG-Thermo Fisher®-(instead of citH3) isotype controls as primary antibodies. After 1h incubation followed by 2 washes with PBS 1x, neutrophils were incubated with secondary antibodies and DAPI. Blue=DAPI staining for DNA; Green=mouse IgG isotype control (to MPO); Red=rabbit IgG isotype control (to citrullinated histone 3); scale bar 24 µm.
