# Supplementary material for: Alkaline pH Promotes NADPH Oxidase-Independent Neutrophil Extracellular Trap Formation: A Matter of Mitochondrial Reactive Oxygen Species Generation and Citrullination and Cleavage of Histone
Source: Front Immunol. 2018 Jan 9;8:1849. doi: 10.3389/fimmu.2017.01849 (PMC5767187; doi:10.3389/fimmu.2017.01849)
Supplement: Supplementary file 14 [file Table_1.PDF]

Table 1. Percentage (%) of dead neutrophils by SYTOX green assay.

-ve control	pH 6.6	pH 7.2	pH 7.8
5 min	0.25 ± 0.11	0.24 ± 0.08	0.56 ± 0.05
10 min	0.32 ± 0.27	0.42 ± 0.22	1.08 ± 0.09
15 min	0.39 ± 0.32	0.50 ± 0.12	1.49 ± 0.10
20 min	0.59 ± 0.40	0.71 ± 0.12	2.27 ± 0.05
25 min	0.74 ± 0.44	1.09 ± 0.32	3.22 ± 0.20
30 min	0.85 ± 0.49	1.13 ± 0.18	4.19 ± 0.73
**A23187**	**pH 6.6**	**pH 7.2**	**pH 7.8**
5 min	0.30 ± 0.07	0.35 ± 0.14	0.48 ±0.08
10 min	0.48 ± 0.39	0.62 ± 0.28	0.96 ± 0.21
15 min	0.75 ± 0.23	0.98 ± 0.44	2.02 ± 1.01
20 min	1.04 ± 0.37	1.78 ± 0.90	2.81 ± 1.10
25 min	1.39 ± 0.54	3.42 ± 2.32	4.44 ± 1.95
30 min	1.59 ± 0.69	5.35 ± 3.08	6.03 ± 2.56
**Ionomycin**	**pH 6.6**	**pH 7.2**	**pH 7.8**
5 min	0.20 ± 0.18	0.32 ± 0.14	0.61 ± 0.12
10 min	0.41 ±0.32	0.73 ±0.34	2.11 ± 1.01
15 min	0.79 ± 0.32	1.36 ± 0.42	6.19 ± 1.20
20 min	1.43 ± 0.40	2.99 ± 0.41	13.74 ± 2.07
25 min	2.19 ± 0.64	5.66 ± 1.01	22.31 ± 2.52
30 min	2.59 ± 0.82	8.16 ± 2.21	28.93 ± 2.60

Table 1. Percentage (%) of dead neutrophils by SYTOX green assay. To confirm that our findings regarding calcium influx and intracellular alkalinization (SNARF assay) after calcium ionophores stimulation were not due to equalization of extracellular and intracellular compartment after cell membrane rupture, we performed a SYTOX green assay, conform described in Figure 1, but plate was read every 5 minutes up to 30 minutes after cells stimulation. Our results confirm that at earlier time points time-points (up to 20 minutes) the neutrophils' viability were higher than 96% and 87% in A23187 and Ionomycin-stimulated cells, respectively. Numbers represent the average of 3 independent experiments (±SEM).
